# Threats to and management of Natura 2000 protected areas relative to agricultural practices

**DOI:** 10.1111/cobi.70172

**Published:** 2025-11-25

**Authors:** Giorgio Zavattoni, Elie Gaget, Tyler Hallman, Ineta Kačergytė, Tomas Pärt, Diego Pavón‐Jordán, Thomas Sattler, Jon E. Brommer

**Affiliations:** ^1^ Department of Biology University of Turku Turku Finland; ^2^ Tour du Valat, Research Institute for the Conservation of Mediterranean Wetlands Arles France; ^3^ School of Environmental and Natural Sciences Bangor University Bangor UK; ^4^ Department of Ecology Swedish University of Agricultural Sciences Uppsala Sweden; ^5^ Birds and Renewables Research Group Norwegian Institute for Nature Research (NINA) Trondheim Norway; ^6^ Swiss Ornithological Institute Sempach Switzerland

**Keywords:** agriculture, agrienvironmental schemes, Common Agricultural Policy, conservation practitioner, ecosystem management, Natura 2000, protected areas, questionnaire, agricultura, áreas protegidas, cuestionario, esquemas agroambientales, manejo de ecosistemas, Natura 2000, practicante de la conservación, Política Agrícola Común, Natura 2000, 保护区, 生态系统管理, 农业, 农业环境计划, 《共同农业政策》, 保护工作者, 调查问卷

## Abstract

The Natura 2000 (N2K) network combines biodiversity protection and socioeconomic targets. Human activities, such as agricultural practices, can affect biodiversity in N2K sites in diverse ways. Limiting activities with negative impacts while enforcing land management that supports biodiversity is crucial for effective conservation. Yet, site‐level information on how this is addressed in N2K sites is lacking. To fill this knowledge gap, we conducted a European Union‐wide survey among N2K site managers. We aimed to assess the implemented conservation measures, their funding sources, and the extent to which different threats are addressed. Of the 341 responses, 61.8% reported the implementation of conservation measures linked to agricultural practices, such as adapting mowing and grazing at levels suitable for the conservation of grassland habitats and species. Sites with management tied to agricultural practices relied more on EU funding, such as the Common Agricultural Policy (CAP), whereas other sites depended more on national funding. Threats not addressed by conservation measures were reported by 63.8% of respondents, suggesting that overall management funding may be insufficient or ineffectively allocated. Most of these unaddressed threats resulted from intensive agricultural practices, such as the use of agrochemicals (reported as a threat in 13% of sites). These findings provide insight into how traditional agricultural practices, mostly related to low‐intensity grazing and mowing, are frequently used as conservation tools, whereas intensive agriculture is a prominent source of unmitigated threats. Thus, achieving N2K conservation goals requires avoiding intensive agricultural practices and strengthening effective conservation measures in protected areas.

## Abstract (es)

Amenazas para y gestión de las áreas protegidas Natura 2000 en relación con las prácticas agrícolas

**Resumen:**

La red Natura 2000 (N2K) combina la protección de la biodiversidad con objetivos socioeconómicos. Las actividades humanas, como las prácticas agrícolas, pueden afectar a la biodiversidad en los espacios N2K de diversas maneras. Limitar las actividades con impactos negativos, al tiempo que se aplica una gestión del suelo que favorezca la biodiversidad, es fundamental para una conservación eficaz. Sin embargo, se carece de información a nivel de sitio sobre cómo se aborda esta cuestión en los sitios N2K. Para llenar este vacío de conocimiento, realizamos una encuesta a nivel de la Unión Europea entre los gestores de sitios N2K. Nuestro objetivo era evaluar las medidas de conservación aplicadas, sus fuentes de financiaciamiento y el grado en que se abordan las diferentes amenazas. De las 341 respuestas, el 61.8% informó de la aplicación de medidas de conservación relacionadas con las prácticas agrícolas, como la adaptación de la siega y el pastoreo a niveles adecuados para la conservación de los hábitats y las especies de los pastizales. Los sitios cuya gestión estaba vinculada a las prácticas agrícolas dependían en mayor medida de la financiación de la UE, como la Política Agrícola Común (PAC), mientras que otros sitios dependían más de la financiación nacional. El 63.8% de los encuestados informó de amenazas no abordadas por las medidas de conservación, lo que sugiere que la financiación global de la gestión puede ser insuficiente o estar asignada de forma ineficaz. La mayoría de estas amenazas no abordadas eran consecuencia de prácticas agrícolas intensivas, como el uso de productos agroquímicos (señalado como una amenaza en el 13% de los lugares). Estos hallazgos proporcionan información sobre cómo las prácticas agrícolas tradicionales, relacionadas principalmente con el pastoreo y la siega de baja intensidad, se utilizan con frecuencia como herramientas de conservación, mientras que la agricultura intensiva es una fuente importante de amenazas sin mitigación. Por lo tanto, para alcanzar los objetivos de conservación de N2K es necesario evitar las prácticas agrícolas intensivas y reforzar las medidas de conservación eficaces en las zonas protegidas.

## 【摘要】

Natura 2000(N2K)网络结合了生物多样性保护和社会经济目标。人类活动(如农业实践)会以各种方式影响N2K保护区内的生物多样性。在N2K保护区内限制具有负面影响的活动, 同时实施支持生物多样性的土地管理措施, 对于其有效保护至关重要。然而, 目前还缺乏有关N2K各个位点如何解决这一问题的信息。为了填补这一知识空缺, 我们对欧盟范围内N2K位点的管理者进行了一次调查, 旨在了解已实施的保护措施、资金来源以及应对不同威胁的程度。在收到的341份回复中, 61.8%的受访者表示实施了与农业实践有关的保护措施, 如调整了割草和放牧的强度以适应草原栖息地和物种的保护。管理涉及农业实践的位点需更多地依赖欧盟资金, 如《共同农业政策》, 而其他位点则更多地依赖国家资金。63.8%的受访者报告了存在保护措施未能解决的威胁, 表明总体的管理资金可能不充足或分配不当。这些未得到解决的威胁大多来自集约化的农业实践, 如使用化学农药(13%的位点将其视为一种威胁)。这些发现使我们深入了解了人们如何常常将传统农业实践(主要表现为低强度放牧和割草)用作一种保护工具, 同时, 集约化农业则是未得到减缓的威胁的主要来源。因此, 要实现N2K的保护目标, 就必须避免集约化农业实践, 并加强保护区的有效保护措施。**【翻译:胡怡思;审校:聂永刚】**

## INTRODUCTION

The establishment of protected areas is recognized as a vital strategy to mitigate the ongoing biodiversity crisis, and international targets aim to protect 30% of the land and sea by 2030 (Chape et al., 2005). However, protected area designation does not in itself ensure conservation success, and ongoing threats require effective management (Gaget et al., 2022; Le Saout et al., 2013; Leverington et al., 2010). In the European Union (EU), the Birds and Habitats Directives (2009/147/EC [EEC, 2009] and 92/43/EEC [EEC, 1992]) provide lists of habitats and species that EU Member States must conserve through the designation of protected areas. The result is an extensive network of protected areas known as the Natura 2000 (N2K), which covers 26.4% of land and 12.1% marine areas of the EU (EEA, 2024a).

The main objective of the N2K network is to maintain and restore habitat and species targets to a favorable conservation status without necessarily excluding human activities. For example, agriculture takes place in 69% of the sites (Tsiafouli et al., 2013), and about 10% of the total N2K area consists of agricultural land (Eurostat, 2021). Article 6(1) of the Habitat Directive states that N2K sites should be managed to reach their conservation goal through the use of targeted conservation measures. Thus, a management plan seems vital, but management plans are not mandatory, and only 62% of all N2K sites currently have one (EEA, 2024b). Funding for the management of N2K sites is the responsibility of the EU Member States, but N2K site management can also be supported by specific EU funding, such as through the EU Programme for the Environment and Climate Action (LIFE+) (Kati et al., 2015).

Despite the strong conservation objectives set by the Birds and Habitats Directives, biodiversity in the N2K network is declining (EC, 2020). An alarming 80% of the habitats listed in the Habitats Directive have an unfavorable conservation status (EC, 2020). National reports point to intensive agriculture (characterized by increased use of inorganic fertilizers, pesticides, and new crop types, including winter crops [Henle et al., 2008; Pain & Pienkowski, 1997; Queiroz et al., 2014; Stoate et al., 2009]) as the main driver for habitat degradation and habitat loss (EC, 2020). Natural areas have been increasingly converted to agricultural land outside and inside N2K sites (Hellwig et al., 2019). Intensive agriculture also negatively affects surrounding natural areas (Kelleghan et al., 2021).

Conservation and agricultural practices are highly interconnected in Europe (Boitani & Sutherland, 2015), where a long history of land cultivation has left the conservation of many species and habitats dependent on traditional agricultural practices, such as low‐intensity grazing and mowing and maintenance of fallow land (e.g., Kruess & Tscharntke, 2002; Ostermann, 1998; Traba & Morales, 2019). However, over the last century, traditional agricultural habitats have declined due to the combined effects of abandonment of less fertile (and less profitable) land and agricultural intensification. Although agricultural intensification typically leads to overall biodiversity loss (Emmerson et al., 2016), abandonment can initiate a community shift and provide an opportunity for the conservation of nonfarmland species (Ceaușu et al., 2015; Sirami et al., 2008).

The EU's Common Agricultural Policy (CAP) has historically facilitated the intensification of agricultural practices and the consequent decline in traditional agricultural habitats and the associated species (Assandri et al., 2019; Emmerson et al., 2016; Pain & Pienkowski, 1997; Reif & Vermouzek, 2019; Traba & Morales, 2019; Wretenberg et al., 2007). However, over time, the CAP has evolved to also include agrienvironmental measures, which provide subsidies for low‐intensity practices (e.g., favoring organic farming) to enhance biodiversity, ecosystem services, and climate change adaptation. Although these schemes can benefit biodiversity, outcomes are variable depending on local and landscape factors (Batáry et al., 2010; Kleijn et al., 2011).

Understanding of the extent to which management of N2K sites can respond to threats is primarily based on information on conservation measures implemented at the level of EU member states. This means that N2K site‐specific information on conservation measures is lacking. The European Commission (EC) collects detailed data on every N2K site. This process is facilitated by the European Environment Agency (EEA), which uses a standard data form (SDF) to gather information on various parameters, such as species targets, importance of the site for the targeted species, and threats. However, the reporting of conservation measures is optional and not standardized (Commission implementing decision 2011/484/EU), despite the existence of a standardized list of conservation measures (Eionet, 2018). The lack of standardization on conservation measures in the SDF limits the EC's ability to use this information to develop evidence‐based policy and reduce the possibility to use these data in conservation studies (Gaget et al., 2022). Local information on conservation management is necessary to improve the effectiveness of biological conservation, but it is challenging to collect (Rodrigues & Cazalis, 2020; Sutherland et al., 2004). Lanzas et al. (2024) recently showed the potential of information on site‐specific conservation measures from N2K sites to be used to identify conservation gaps and support decision‐making. By collecting standardized information on conservation measures at a protected area level, they highlighted spatial management gaps at regional level that can guide a more informed conservation planning process.

We conducted an EU‐wide survey of a representative sample of N2K site managers to gather site‐specific information on conservation measures, threats, and funding used. We obtained an overview of the frequency of different conservation measures implemented across the network and the threats they address. As agricultural practices have been highlighted as an important cause of current biodiversity declines, even in N2K sites, we also determined the following: the proportion of site managers who considered the implementation of conservation measures related to agriculture and agricultural habitats to be the most important strategy for biodiversity conservation and whether this proportion varies between N2K sites characterized by different habitat types; which threats posed by agricultural practices are being addressed (i.e., addressed threats) by conservation measures and which are not (i.e., unaddressed threats); which threats are posed by agricultural abandonment and which are posed by intensive practices; and how the use of EU funding to implement management differed between sites relying on agriculture‐related strategies and sites relying on other conservation measures.

We hypothesized that the most important conservation measures are frequently tied to agricultural practices and that in N2K sites, and where this is the case, management relies more on EU funding (such as the CAP) than in other sites.

## METHODS

### Survey structure

To collect information on the management of N2K sites, we conducted a survey targeting N2K managers. The survey asked the managers to select an N2K site, specify whether they managed the whole site or part of it, and identify additional spatially overlapping N2K sites managed as a single protected area with the first selected N2K site. If a manager was responsible for more sites in an area, they were asked to complete the survey for as many sites as they could, starting with the ones they personally judged to be more representative of the area. Next, the survey asked N2K site managers to provide a list of all conservation measures implemented in the selected site over the last 5 years and to identify which of these measures they considered the most important for protecting the site's biodiversity. They were also asked to indicate and rank the relative importance of funding sources: public funding (e.g., national, regional, county), LIFE+, other EU funding (including CAP), private funding (e.g., companies, foundations), and other (e.g., crowdfunding, volunteering). Finally, the survey asked respondents to specify which threats to biodiversity were not addressed by any conservation measures (hereafter simply referred to as unaddressed threats). This question allowed us to examine unmanaged threats to N2K sites. The survey was built interactively from publicly available information in the SDFs, so the list of possible answers to certain questions depended on the data in the SDFs of the selected site or sites. For example, when the survey asked the respondent to select which threats were not addressed by any conservation measures, the list of possible answers was the list of threats provided in the SDF for the selected site, making the completion process faster and more user friendly. Respondents were given the option to manually update the SDF information if it was deemed incorrect or incomplete. The complete questionnaire is in Appendix S1. The survey was built in R.4.2.1 (R Core Team, 2022) with the shiny package and hosted on the Shinyapp platform (Chang et al., 2022).

### Conservation measures and threats

The data on conservation measures and threats we collected in the survey were based on a classification of the EEA. The list has 105 different conservation measures divided into 12 broad categories (Eionet, 2018) and 400 threats divided into 13 categories (Eionet, 2011). For the sake of simplicity, we used the same categories but merged 3 different threats (natural system modifications, natural biotic and abiotic processes, and geological events and natural catastrophes) into one category: natural systems and processes. By doing so, each conservation measure category could be matched to a threat category, except for “measures related to management of species from the nature directives and other native species” (Table 1) (Eionet, 2011, 2018). This category is very broad because it includes generic actions without a specified target, such as “improvement of habitat of species from the directives” (CS03).

**TABLE 1:** Threats to Natura 2000 protected areas and conservation measures as described by the European Environment Agency and their assigned category.

Category	Threat	Conservation measure^a^
Agriculture	Agriculture	Measures related to agriculture and agriculture‐related habitats
Forestry	Sylviculture, forestry	Measures related to forestry and forest‐related habitats
Resource extraction	Mining, extraction of materials, and energy production	Measures related to resources extraction and energy production
Transports	Transportation and service corridors	Measures related to development and operation of transport systems
Urbanization	Urbanization, residential and commercial development	Measures related to residential, commercial, industrial, and recreational infrastructures, operations, and activities
Species exploitation	Biological resource use other than agriculture and forestry	Measures related to the effects of extraction and cultivation of biological living resources
Human disturbance	Human intrusions and disturbances	Measures related to military installations and activities and other specific human activities
Problematic species	Invasive, other problematic species and genes	Measures related to non‐native and problematic native species
Pollution	Pollution	Measures related to mixed source pollution and human‐induced changes in hydraulic conditions for several uses
Natural systems and processes	Natural system modifications, natural biotic and abiotic processes, geological events, natural catastrophes	Measures related to natural processes, geological events, and natural catastrophes
Climate change	Climate change	Measures related to climate change
Species‐specific management	–	Measures related to management of species from the nature directives and other native species^a^

^a^ Could not be matched with any threat category.

To explore the different roles of land abandonment and agricultural intensification in the management of N2K sites, we further divided agricultural conservation measures and threats into different categories. We did so deductively by reading their definitions provided by the EEA (Eionet, 2018). We separated conservation measures related to agriculture into 3 categories: measures preventing agricultural practices, measures supporting agricultural practices, and measures of unspecified type related to agriculture (Appendix S2). For example, we classified the conservation measure “prevent conversion of natural and semi‐natural habitats, and habitats of species into agricultural land” (CA01) in measures preventing agricultural practices; the conservation measure “reinstate appropriate agricultural practices to address abandonment, including mowing, grazing, burning, or equivalent measures” (CA04) in measures supporting agricultural practices; and the conservation measure “other measures related to agricultural practices” (CA16) in measures of unspecified type related to agriculture. Similarly, we separated agricultural threats into threats related to the presence of agricultural practices, threats related to the abandonment of agricultural practices, and threats of unspecified type related to agriculture (Appendix S3). For example, we included the threat “grassland removal for arable land” (A02.03) in threats related to the presence of agricultural practices; the threat “abandonment of pastoral systems, lack of grazing” (A04.03) in the threats related to the abandonment of agricultural practices; and the threat “annual and perennial non‐timber crops” in the threats of unspecified type related to agriculture A06.

To visualize what was the most important conservation measure in N2K sites characterized by different habitats, we referred to the classification of habitat classes reported for each site in the SDF (EEA, 2021), which we further categorized into 3 broader categories (agricultural and grassland, forest, and other) (Appendix S4). We then separated the sites according to which of the 3 broad habitat categories had the highest spatial coverage.

### Survey distribution

The survey was translated by native speakers into all EU languages (except for Romanian, Danish, and Estonian, for which only the English version was available) and distributed between 1 November 2023 and 15 March 2024. An invitation to participate in the survey was sent to all the contacts available on the SDFs as reference for the management of the sites and to all N2K national coordinators listed on the EEA website (Eionet, 2023). In addition, through a collaboration with the European nongovernmental organization (NGO) Eurosite, we were able to disseminate the survey to N2K site managers in the EU who were members of the NGO. Together with Eurosite, we also organized a webinar with the Directorate‐General for Environment (DG ENV) to promote the survey. Finally, we searched the internet for additional people to invite to participate in the survey.

### Statistical analyses

Statistical analyses were performed using R.4.2.1 (R Core Team, 2022). To assess the representativeness of the sampled N2K sites compared with the whole N2K network, we used a chi‐square test for given probabilities. We compared the distribution of the surveyed N2K sites with the whole N2K in terms of bioregions, their size, and reported threats according to the SDF information.

Generalized linear models (GLMs) were used to test whether the prevalence of implemented agricultural conservation measures was higher across N2K sites than conservation measures of other categories; whether the prevalence of addressed and unaddressed agricultural threats was higher across N2K sites than other categories of threats; and whether sites that relied on agriculture‐related management had different funding sources than other sites. To answer the first question, we used the presence or absence of conservation measures as a response variable and the measure's category (e.g., agriculture, forestry) as the explanatory variable. Similarly, for the middle 2 questions, we used the presence or absence of addressed and unaddressed threats, respectively, as response variables and threat category as the explanatory variable. To answer the last question, we ran separate GLMs for each of the predominant funding sources (EU including CAP, state funding, LIFE) with data on the presence or absence of the funding source as the response variable and binary data on sites with or without the main conservation measure related to agriculture as the explanatory variable (binomial error distribution with loglink). The models were run accounting for spatial dependencies in the residuals thanks to a Gaussian random field built with a spatial mesh created from site coordinates with a cutoff distance of 2 decimal degrees (sdmTMB package) (Anderson et al., 2024). The absence of spatial autocorrelation was then validated using the DHARMa package (Hartig, 2022). Specifically, we assessed autocorrelation based on the Moran's *I* with the function testSpatialAutocorrelation and an alpha risk of 5%. The *p* values were extracted using the emmeans package (Lenth, 2022).

Code and data are available in Appendix S12.

## RESULTS

**FIGURE 1:**
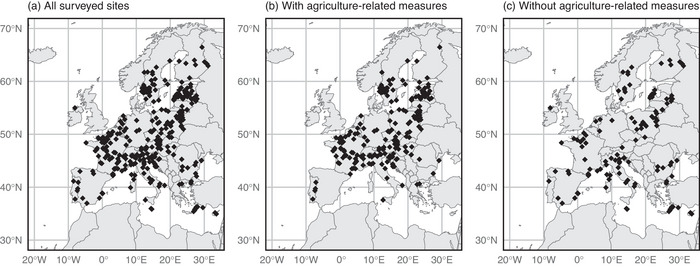
Distribution of the Natura 2000 (N2K) sites (a) with data on management (single location shown for multiple spatially overlapping sites [*n* = 341]), (b) that managers reported as having at least one conservation measure related to agricultural practices, and (c) that were not reported as having conservation measures related to agricultural practices.

We received 341 unique answers from 25 different countries (Figure 1) that documented conservation measures implemented at 505 N2K sites covering an area of 116,085 km^2^ (2.4% of EU's land area) from 2018 to 2023. When one answer included multiple overlapping sites, these were treated as one unit in the analyses. The surveyed N2K sites represented a generally well‐balanced sample of the entire N2K network in terms of bioregions and reported threats, though with some minor discrepancies (Appendices S5a & S5b). A more significant bias was present in relation to the site size because we received more responses from large sites (Appendix S5c).

### Overview of conservation measures and threats

**FIGURE 2:**
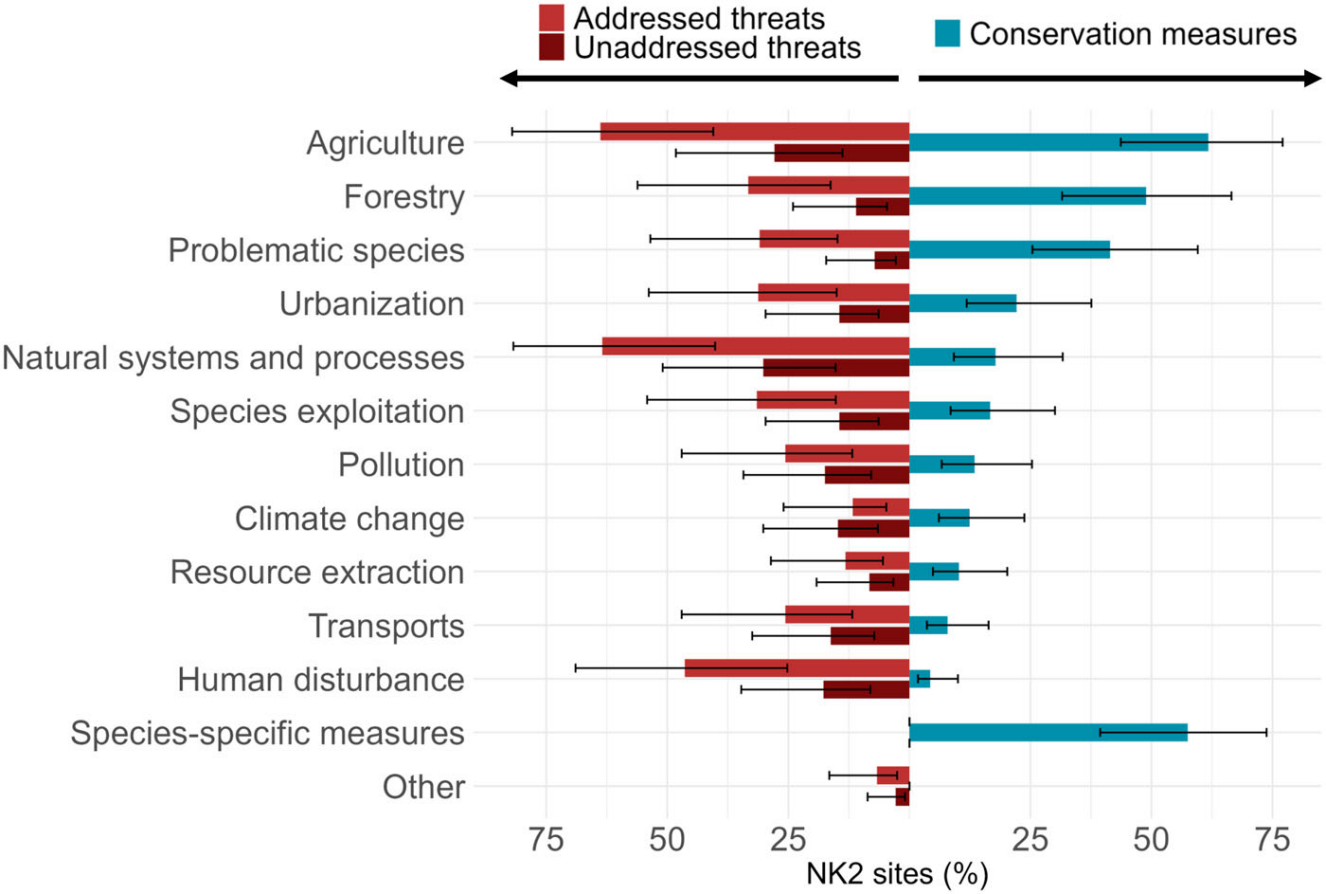
Predicted percentages (95% CI) of Natura 2000 sites (N2K) where at least one conservation measure has been carried out (right) and where at least one threat has been addressed (left) and unaddressed (left) by any conservation measure by categories of the European Environment Agency threat and measures classification scheme. Predicted percentages are from a generalized linear model and are shown instead of the raw data to better account for spatial autocorrelation.

Conservation measures related to agriculture (estimated in 61.8% [95% CI 43.7–77.1] of sites) were one of the most widespread measures in the N2K sites surveyed, although they were statistically similar to values for forestry and species‐specific measures (Figure 2) (see Appendix S6 for comprehensive pairwise model results). The total number of unaddressed threats (threats identified by managers currently not counteracted by any conservation measures) accounted for 27.0% of all reported threats. About 93.6% of the N2K sites in our survey had at least one threat addressed by conservation measures, whereas 63.8% of the sites had at least one unaddressed threat. Agriculture was the main source of addressed threats (estimated at 63.8% [95% CI −40.5 to 82.1] of N2K sites) together with natural systems and processes (estimated at 63.4% [95% CI 40.1–81.8]). The category natural systems and processes was also the primary source of unaddressed threats (estimated in 30.2% [95% CI 15.2–50.9] of N2K sites), followed by agriculture (estimated in 27.9% [95% CI 13.8–48.2] of N2K sites) (see Appendices S7 & S8 for comprehensive model results of pairwise comparisons). Respondents selected 2546 currently unreported threats in their SDF across 145 out of the 341 N2K sites; on average, each site had 7.5 unreported threats (median = 0 [SE 0.9]). Unreported threats accounted for 42% of the total threats, highlighting large gaps in the existing SDF information (see Appendix S9 for the distribution of unreported threats across the different categories defined by the EEA).

### Most important conservation measures

**FIGURE 3:**
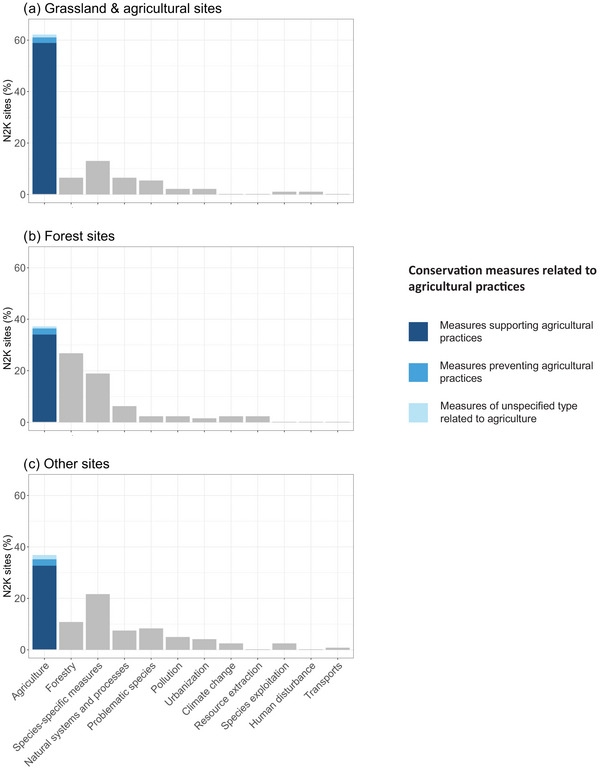
Percentages of Natura 2000 (N2K) sites by their primary conservation measures grouped by the dominant habitat type: (a) grassland and agricultural, (b) forest, and (c) other; and conservation measures related to agricultural practices (supporting agricultural practices, e.g., mowing and grazing; preventing agricultural practices, e.g., prevention of mowing, grazing, and conversion of natural areas to agricultural).

Conservation measures related to agriculture were considered the most important measures employed for biodiversity conservation in nearly half (44.8%) of all N2K sites managed by survey respondents. Not surprisingly, conservation measures related to agriculture were most important in N2K sites dominated by grassland and agricultural habitats (63.3% of these sites) (Figure 3a). However, these agriculture‐related conservation measures were also the most prominent actions in N2K sites dominated by forest (37.6% of these sites) (Figure 3b) and N2K sites dominated by other habitats (37.2% of these sites) (Figure 3c). More specifically, in agriculture‐related important conservation measures, almost all (93.2%) supported nonintensive agricultural practices, such as mowing and grazing. In contrast, only a few (4.8%) opposed agricultural practices, for example, by stopping grazing and mowing or by preventing the conversion of natural habitats into agricultural land (Figure 3; Appendix S10 contains details on the frequency of each conservation measure).

### Unaddressed agricultural threats

**FIGURE 4:**
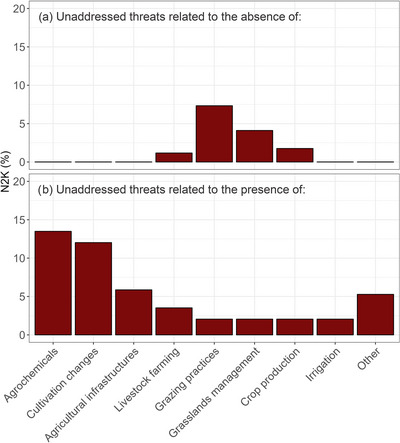
Percentage of Natura 2000 (N2K) sites with different unaddressed threats to biodiversity relative to the (a) absence of agriculture and (b) presence of agriculture. The threat categories are those of the European Environment Agency.

Among the agriculture‐related unaddressed threats, the use of agrochemicals was the most common (13.5% of sites), followed by changes in cultivation practices (12.0% of sites) (Figure 4b). In many N2K sites, grazing practices were also reported to be a problem. The absence of grazing was a threat in 7.3% of sites, and the presence of grazing was a threat in 2% of sites (Figure 4). Overall, 10% of N2K sites had at least one unaddressed threat related to the absence of agricultural practices, and 21.7% of sites had at least one unaddressed threat related to the presence of agricultural practices.

### Management funding

**FIGURE 5:**
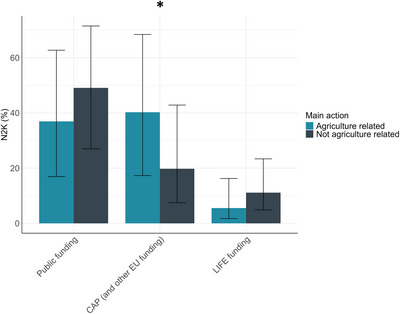
Primary funding sources for management of Natura 2000 sites (N2K) relative to whether the main conservation measure is agriculture related or not (*, pairwise differences statistically significant at *p* < 0.05; CAP, Common Agricultural Policy; LIFE, Programme for the Environment and Climate Action). Percentages (95% CI) are predicted based on a general linear model.

There was a difference in the primary funding sources used (Figure 5): N2K sites where agriculture‐related measures were the most important for biodiversity tended to rely twice as much on the CAP and other EU funding as N2K sites where measures were not primarily related to agriculture (*p* < 0.01). For the latter category, funding came predominantly from public funding (state, region, county funding), although not statistically significantly more than for sites where agriculture‐related measures were the most important for biodiversity (*p* = 0.11) (comprehensive results in Appendix S11).

## DISCUSSION

Our site‐level results highlighted the overarching impacts of agricultural intensification and abandonment on the N2K network of protected areas and are consistent with results of previous studies at the national level that show a high number of overall unaddressed threats across the N2K network (Hermoso et al., 2022). Consistent with our original hypothesis, we found that some of the most frequent and important actions (according to the managers’ opinions) implemented in N2K sites belonged to the EEA category of conservation measures related to agriculture. These measures mainly focus on maintaining open areas through traditional farming practices involving adapted mowing and grazing. In contrast, a major source of unaddressed threats is the presence of intensive agricultural practices. These findings are in line with those of the EC's State of Nature report (EC, 2020), which also identified agricultural intensification and land abandonment as major threats to N2K sites, but this report was based on only national data.

Responses from N2K site managers indicated that the information stored in the SDF is not being properly updated. In particular, managers identified several threats not listed in the SDF. Relying only on N2K data stored by the EEA for conservation assessments risks misidentifying threats within sites. This finding complements studies that show the information on the SDFs is incomplete in terms of the species present at a site (Lisón et al., 2017). These are critical problems given that the purpose of the SDFs is to assess the effectiveness of the N2K network, decide on funding allocations, facilitate decision‐making processes, and provide a reference source for assessing specific issues in case of potential violations of European environmental law (EC, 2011).

### Management funding

Overall, our results possibly suggest that the overall funding available to manage N2K sites may not be sufficient or effectively allocated, given the high frequency of unaddressed threats reported. The EU CAP funding was the main support for managing N2K relying on conservation measures related to agriculture, whereas other N2K sites depended more on national funds. The inadequacy of national‐level funding is of particular concern given how the recently adopted Nature Restoration Law requires Member States to provide the funding to carry out the required restoration efforts, though they can still be co‐supported by EU funding (European Parliament, 2024). These efforts are expected to be added to the resources already necessary for the current everyday management of N2K sites.

Our results underline how much management of N2K sites, especially the ones dealing with agricultural practices, relies on the CAP to carry out conservation measures. If CAP is a critical extra source of funding, the CAP‐funded measures may not be effective enough compared with more specific management strategies (Batáry et al., 2015), and they may not fully benefit non target species (Gaget et al., 2019). There have even been reported cases where CAP‐funded measures in N2K damaged the very biodiversity they were meant to protect. For example, the intensity of grazing required to receive CAP subsidies for some N2K sites in Sweden was too high for one target species, the marsh fritillary (*Euphydryas aurinia*) (Kindvall et al., 2022). This suggests that, although CAP funding is crucial for N2K sites, there is an urgent need to ensure that beneficial agricultural practices are prioritized over those that can be detrimental to biodiversity. Importantly, the CAP can also provide funding to set land aside from agricultural activity, and this is likely the most effective measure for conservation (Batáry et al., 2015).

We also found that EU LIFE+ was rarely the most important source of funding under any circumstances. However, LIFE‐funded actions have generally been judged more positively for biodiversity conservation than CAP‐funded actions (Kati et al., 2015), although they are insufficient and biased toward some regions and some taxa (Hermoso et al., 2017; Mammola et al., 2020). The LIFE+ budget (€5.4 billion for 2021–2027 [EC, 2021a]) is dwarfed by the expenditure on CAP (€386.6 billion for 2021–2027), of which €95.5 billion are allocated to the second pillar, which supports low‐intensity agricultural practices (EC, 2021b).

### Agriculture and conservation in Europe

Most European grasslands are seminatural and formed and maintained through traditional mowing or grazing (Assandri et al., 2019). Although partially sustained by human activities, these are among the most biodiversity‐rich habitats in Europe (Habel et al., 2013). Heterogenous habitats with open areas were once maintained by wild herbivore megafauna, but since the extinction of many of these natural grazers, traditional farming practices, especially grazing and mowing, have become a somewhat artificial substitute (Boitani & Sutherland, 2015; Pykälä, 2000; Sutherland et al., 2004). The use of livestock to replicate the impact of lost large herbivores is being increasingly recognized by the rewilding movement (Gordon et al., 2021), and grazing and mowing are often implemented as routine management measures, sometimes even inside strictly protected areas (Boitani & Sutherland, 2015). Our results reflect this seemingly paradoxical situation in Europe, where humans have been affecting the environment for so long that many natural systems and processes no longer exist and biodiversity often depends on human disturbance (Boitani & Sutherland, 2015; Ellis et al., 2021). Therefore, if a similar survey was conducted in other parts of the world, the results would likely be different, possibly showing conservation efforts more focused on maintaining natural and wilderness areas.

The importance of human‐maintained habitats for biodiversity in Europe is also highlighted in the Habitats Directive, where 27% of the listed habitat types are dependent on (10%) or can benefit from (17%) low‐intensity agricultural practices, including pasture maintenance and grazing, haymaking, and crop growing (Halada et al., 2011). Many of these habitats, though not all, are categorized as grasslands in the Habitat Directive, which alone cover around 16% of the total area of Annex I habitats (EC, 2020). Additionally, habitats relying on traditional low‐intensity agricultural practices are on average in poorer conservation condition relative to other directive habitats (EC, 2020; Halada et al., 2011). Therefore, our finding that conservation measures are so often linked to agricultural practices, even in N2K sites dominated by habitat types other than grassland and agricultural land, may be explained by 2 factors: the disproportionate importance of these open areas for biodiversity and their current poor condition and the need for human intervention to maintain their state. Although the State of Nature Report showed that 80% of the directive habitats have an unfavorable conservation condition (EC, 2020), many of these may benefit more from increased protection and passive rather than active interventions. The recently adopted Nature Restoration Law (European Parliament, 2024) recognizes how certain areas can naturally recover by simply stopping or limiting some of the existing pressures.

### Limitations

We collected information on implemented conservation measures, but we were unable to assess their effectiveness. Measuring the effectiveness of conservation interventions is crucial to improve and guide future management decisions, but the effects of management are not always measured (Sutherland et al., 2004). We also asked protected areas managers to select the most important conservation measure at their site, based on their personal opinion. The influencing factors for this choice could be based on the number of targets addressed, the importance of the counteracted threats, or some other subjective consideration. Although expert opinion can offer valuable insights into context‐specific management practices, perceptions of what are important conservation measures and threats to biodiversity can be partly influenced by personal experience (Sutherland et al., 2004). Limited conservation means and resources could also induce conservation trade‐offs at the local scale that result in management gaps and suboptimal conservation efforts at large scale (Lanzas et al., 2024).

Our results showed that collecting management information at the site level is possible, though challenging. Despite considerable efforts in communication, we received responses from only 1.8% of the sites in the N2K network. The limited number of responses in our survey may be at least partially explained by insufficient staff of national organizations that manage N2K sites and insufficient resources to complete the survey. Our survey, however, appears representative of the overall N2K state, as the collected data showed no clear biases, except from a bias toward larger sites. This bias may result from managers responsible for multiple sites choosing a larger site they considered representative or relevant when completing the survey. Larger N2K sites may include a mosaic of different habitats and land uses, which can be characterized by different management strategies. In contrast, smaller sites may be more homogenous and less exposed to human activities and thus require less management. Therefore, our survey results may be skewed toward threats and conservation measures associated with large and more managed N2K sites. It is also possible that managers in charge of several sites may have selected the best‐managed areas to show what they are doing or the most degraded areas to emphasize the critical situation for biodiversity. Despite the biases, the 341 responses to our study provide a valuable overview of the threats facing N2K sites and how managers are dealing with them. Site‐specific information on conservation measures, threats, and targets is needed to assess conservation effectiveness and are thus of value for evidence‐based conservation and decision‐making. Information on conservation measures should be added to the SDF to allow in‐depth conservation assessments.

We focused on management carried out inside N2K sites; however, the effectiveness of conservation efforts in these areas is influenced by threats coming from surrounding landscapes (Palomo et al., 2014). Worryingly, land‐use changes in the immediate surroundings of N2K sites have been happening faster than in other unprotected areas in Europe (Hellwig et al., 2019). Increased land‐use changes, such as farming practices resulting from agricultural mechanization, happening outside protected areas are contributing to their isolation and can disrupt ecological functions and increase extinction risks in them (Hansen & DeFries, 2007; Woodroffe & Ginsberg, 1998). To address this, and to compensate for the often‐limited coverage of protected areas, the importance of carrying out management measures in unprotected sites to reach conservation targets is being increasingly recognized (Rodríguez‐Rodríguez et al., 2021).

### Agricultural unaddressed threats

Our findings showed that livestock grazing is one of the most widely implemented conservation measures and N2K managers often selected it, according to their personal opinion, as the most important measure for biodiversity conservation in their sites. However, grazing is also an unaddressed threat in many N2K sites because of both overgrazing and undergrazing. Grazing is a recurring topic in conservation discussions in Europe because when done extensively, it is thought to mimic grazing by large wild herbivores that are now mostly extinct on the continent (Schowanek et al., 2021). However, extensive grazing by cattle, horses, goats, and sheep has declined following changes in agricultural practices in recent decades, including mechanized farming, motor vehicles, intensified meat production, and rural depopulation (Collantes, 2009; Haddad et al., 2019; Jepsen et al., 2015). Conversely, our results also highlight instances where overgrazing is a threat to biodiversity in N2K sites. Grazing can threaten biodiversity when it is intensified or poorly managed, leading to habitat degradation and altered plant community dynamics, which can ultimately cause biodiversity loss (Bröder et al., 2019; Kindvall et al., 2022; Varga et al., 2021). The effects of grazing are therefore highly context dependent, and grazing must be carefully evaluated when it is used as a conservation tool (Bussan, 2022; Sartorello et al., 2020; Török et al., 2024).

Our results also highlighted the widespread concern among N2K managers about the use of agrochemicals (i.e., pesticides and fertilizers). Despite their well‐documented negative impacts on biodiversity (e.g., Dudley & Alexander, 2017; Geiger et al., 2010; Wan et al., 2025), the use of agrochemicals is permitted inside some N2K sites (Möckel, 2022), and they can easily diffuse from surrounding areas and contaminate protected sites (Kelleghan et al., 2021; Liess et al., 2021; Merleau et al., 2024). Changing policies that regulate the use of agrochemicals inside and outside N2K sites should therefore be a priority.

### Other threats

Agricultural intensification is just one of the many challenges of N2K management that survey respondents noted. Even though it was the most prevalent challenge mentioned, the sum of all other stressors for biodiversity was more significant. Not surprisingly, forestry‐related measures were also widespread. As with agriculture, forestry practices are allowed inside protected areas, occurring in about 59% of N2K sites (Tsiafouli et al., 2013). Many conservation measures are also taken to manage invasive and native problematic species, which have been previously reported to affect several different habitats across the N2K network (Guerra et al., 2018; Lazzaro et al., 2020; Perzanowska et al., 2019), and to manage the impacts of urbanization. Urbanization is a growing concern inside the N2K network, as highlighted in the State of Nature Report (EC, 2020). Higher urban growth rates have been recorded in N2K sites than in unprotected areas, suggesting that proper management plans are necessary but often not implemented strongly enough to halt urban sprawl (Concepción, 2021).

One of the primary sources of threats—addressed and unaddressed by conservation measures—is natural system and process modifications, although this category is broad and encompasses many issues, including some closely tied to farming practices. One of these issues is water abstraction for agricultural purposes, which is a detrimental aspect of agricultural intensification and a major driver of wetland loss (Acreman & Salathe, 2022; Taylor et al., 2021). Another notable source of threats is direct human disturbance, which conservation measures rarely counteract. This category mostly includes issues related to tourism activities, which are a commonly debated topic in conservation. Tourism creates economic benefits for N2K sites, promotes their acceptance, and increases environmental awareness, but it can greatly damage landscapes and disturb wildlife if not properly managed (Belsoy et al., 2012; Rocchi et al., 2020).

### Policy implications

Our findings provide the N2K site managers’ perspective on the ongoing debate about agricultural intensification and conservation in Europe, which is particularly relevant in today's political context. In December 2019, the EU launched the Green Deal, a set of policy initiatives that included a set of ambitious targets to address the deleterious effects of intensive agriculture on biodiversity, as detailed in the Farm to Fork and Biodiversity Strategy for 2030 (EC, 2019). Specific targets mentioned in the original strategies included a 50% reduction in the use of chemical pesticides by 2030; restoring at least 10% of agricultural areas with high‐diversity landscape features (e.g., buffer strips, rotational or nonrotational fallow land, hedges, non productive trees, terrace walls, and ponds); and having 25% of all agricultural land being farmed organically (EC, 2019). The Green Deal was accompanied by what was advertised as a greener revision of the CAP for 2023–2027 (EC, 2021c), although this has been judged as less ambitious for biodiversity conservation and climate change mitigation than the Green Deal (Guyomard et al., 2023). Nonetheless, both proposals have been met with widespread resistance from part of the farming community, which expressed concerns over the risk of economic losses and increased bureaucracy. These frustrations culminated in massive protests across Europe in the spring of 2024, coinciding with the period of our survey. In response, the EU stepped back and loosened several planned environmental regulations. For instance, the Green Deal's original goal of reducing pesticide use by 50% by 2030 was dropped, and a mandatory CAP requirement to set aside 4% of farmland for biodiversity was also retracted (Chapron, 2024).

Regardless of the resistance expressed in the farming protests, our results add further evidence of the importance of implementing adequate policies regulating farming practices inside and outside protected areas for biodiversity conservation in Europe. It will be highly challenging to achieve EU conservation goals without addressing these practices given their pervasiveness even in protected areas. Priorities should include, for example, reinstating the pesticide reduction proposal (Bosco et al., 2024) and limiting CAP subsidies for intensive practices in favor of practices that support extensive and organic farming, which should be designed to meet locally specific conservation needs. Additionally, more funding for conservation, such as the LIFE+ or national funding, could also help promote management measures that are decoupled from agricultural production and focused exclusively on biodiversity conservation.

## Supporting information



Supplementary Material

Supporting data for running the code

Supporting data for running the code

Supporting data for running the code

Supporting data for running the code

Supporting data for running the code

Supplementary Material
