# Supplementary material for: Threats to and management of Natura 2000 protected areas relative to agricultural practices
Source: Conserv Biol. 2025 Nov 25;40(2):e70172. doi: 10.1111/cobi.70172 (PMC13036295; doi:10.1111/cobi.70172)
Supplement: Supplementary file 1 — Supplementary Material [file COBI-40-e70172-s007.docx]

**Supporting information for “Threats to and management of Natura 2000 protected areas relative to agricultural practices”**

Table of contents

[**Appendix S1** 2](#_Toc215061309)

[**Appendix S2** 14](#_Toc215061310)

[**Appendix S3** 15](#_Toc215061311)

[**Appendix S4** 18](#_Toc215061312)

[**Appendix S5** 20](#_Toc215061313)

[**Appendix S6** 21](#_Toc215061314)

[**Appendix S7** 24](#_Toc215061315)

[**Appendix S8** 27](#_Toc215061316)

[**Appendix S9** 30](#_Toc215061317)

[**Appendix S10** 31](#_Toc215061318)

[**Appendix S11** 33](#_Toc215061319)

**Appendix S1**. Screenshots of the online survey for Natura 2000 site managers.

**
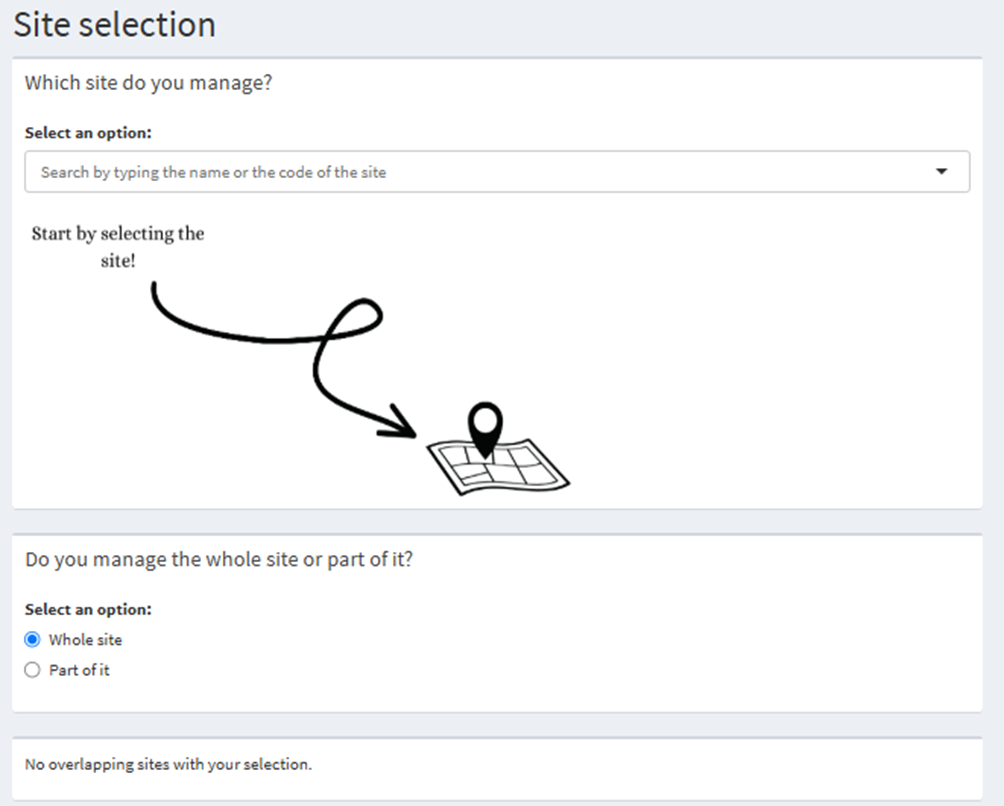
**

**
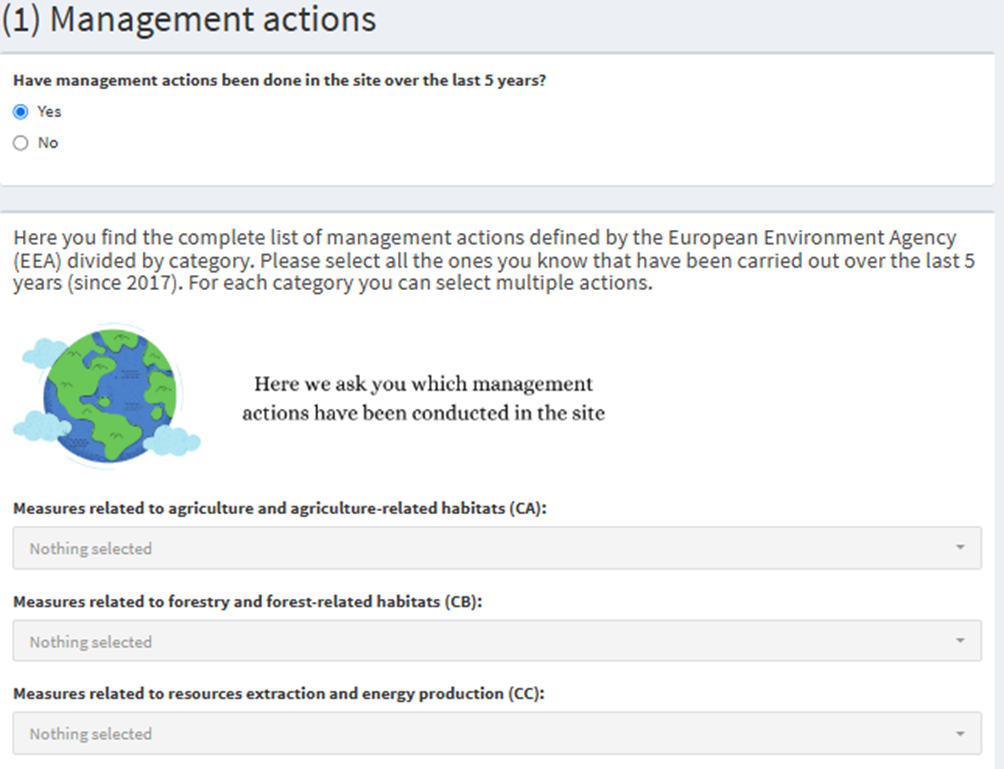
**

**
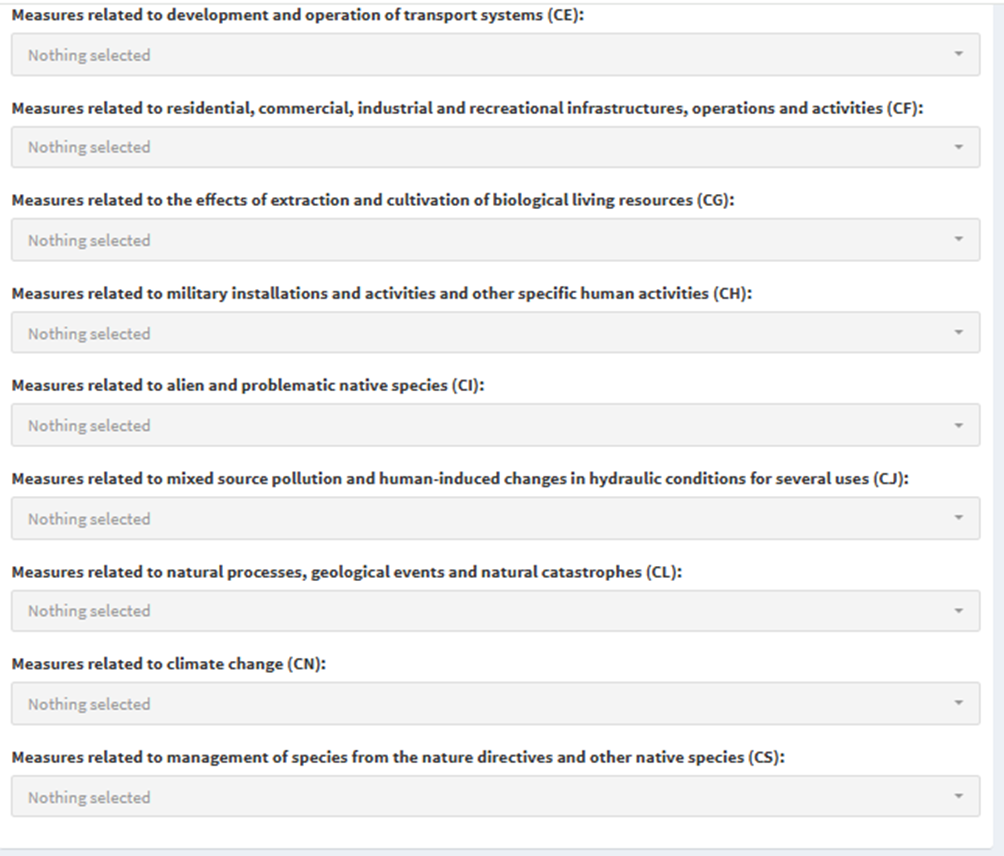
**

**
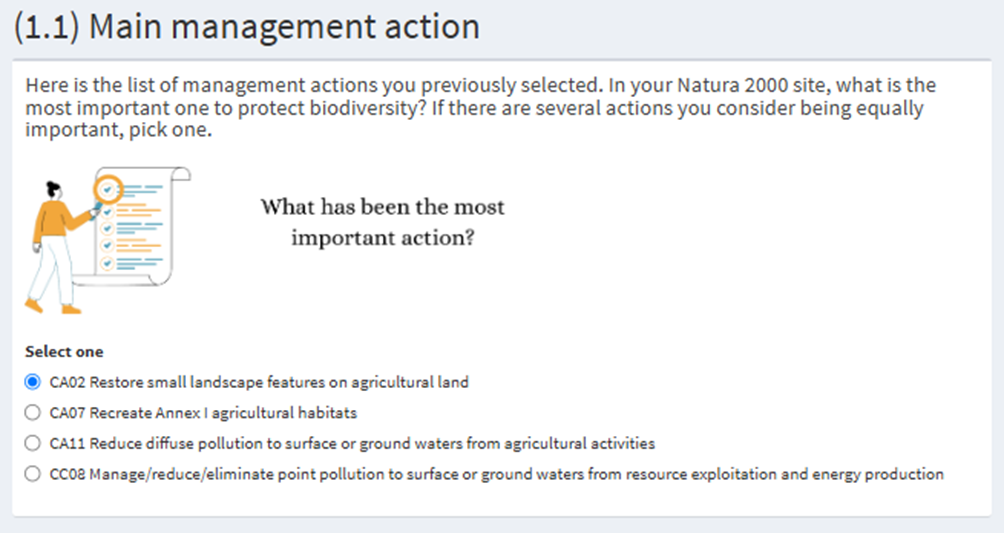

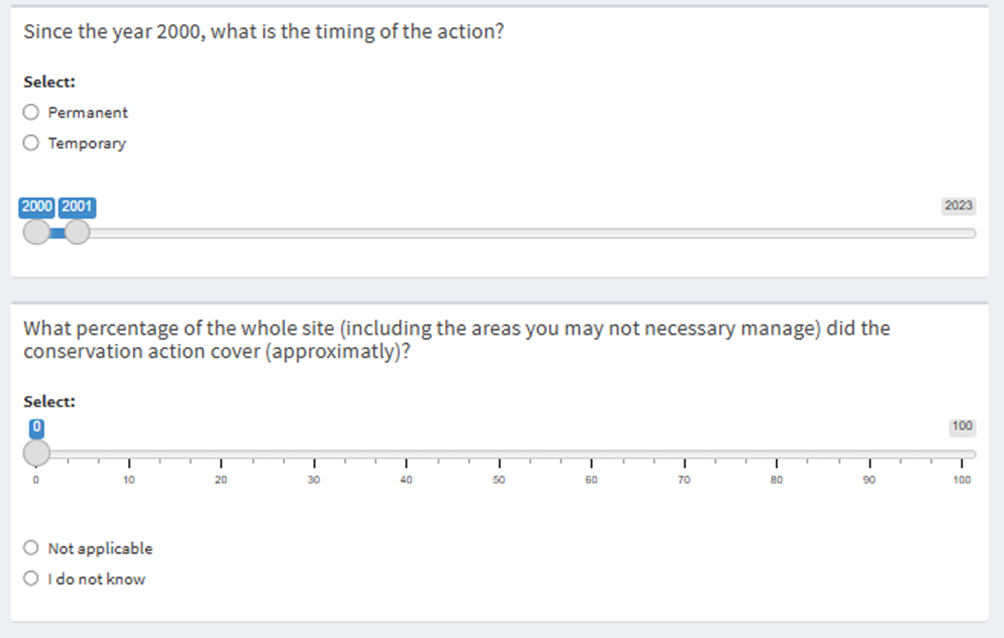

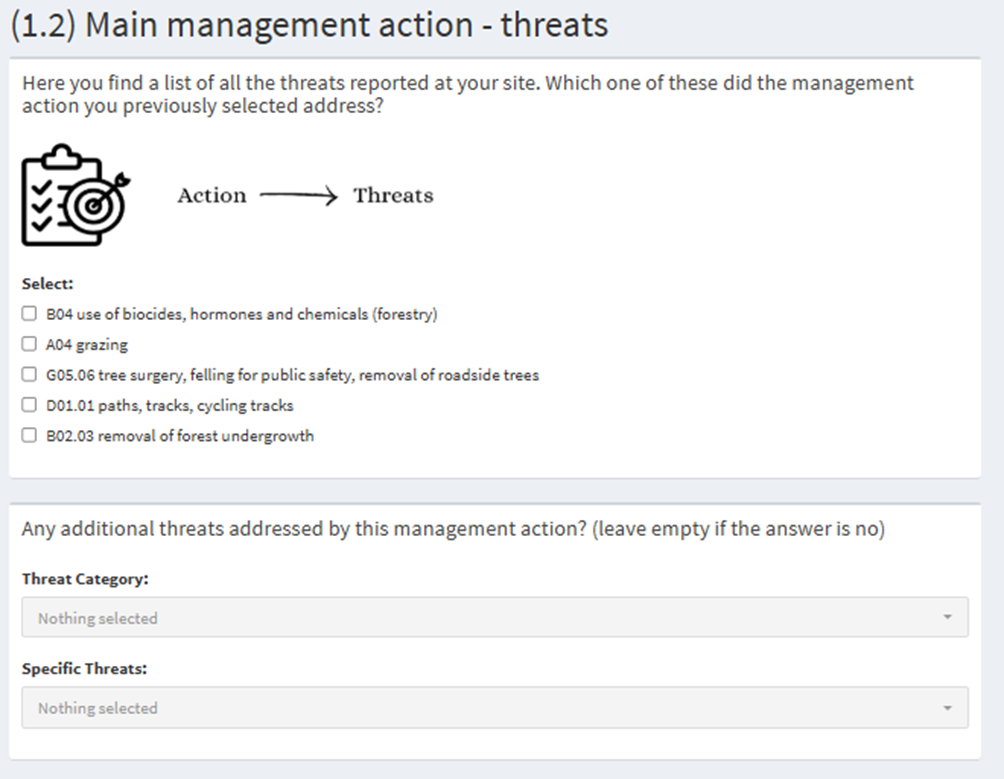

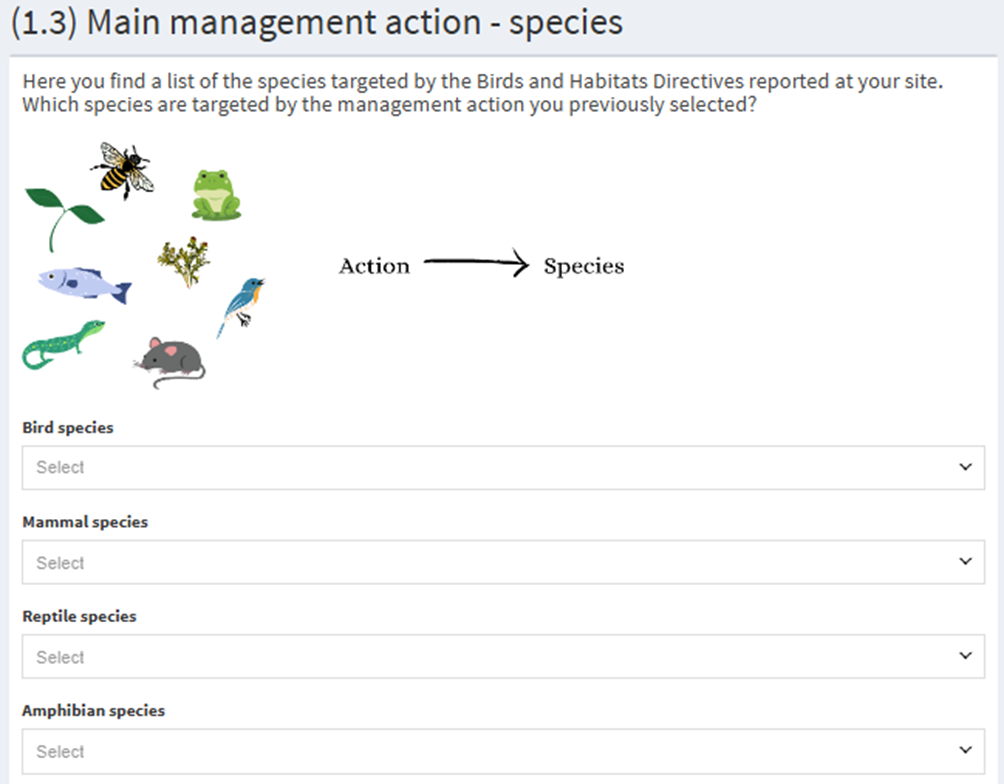

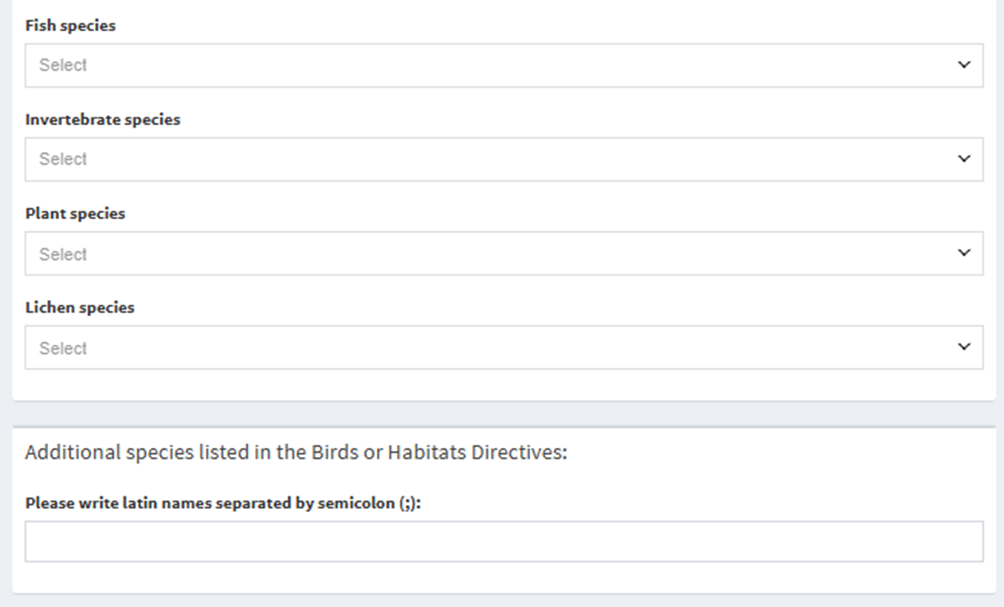

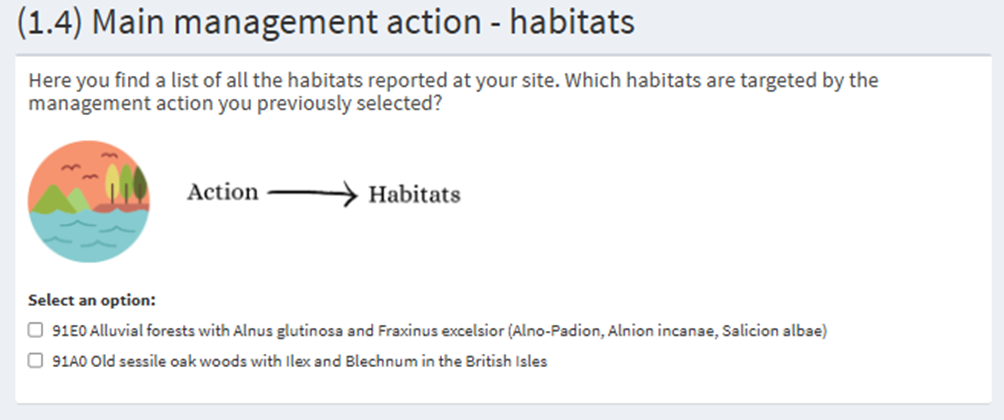

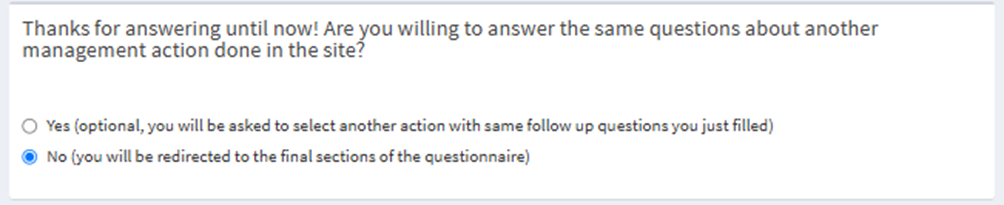

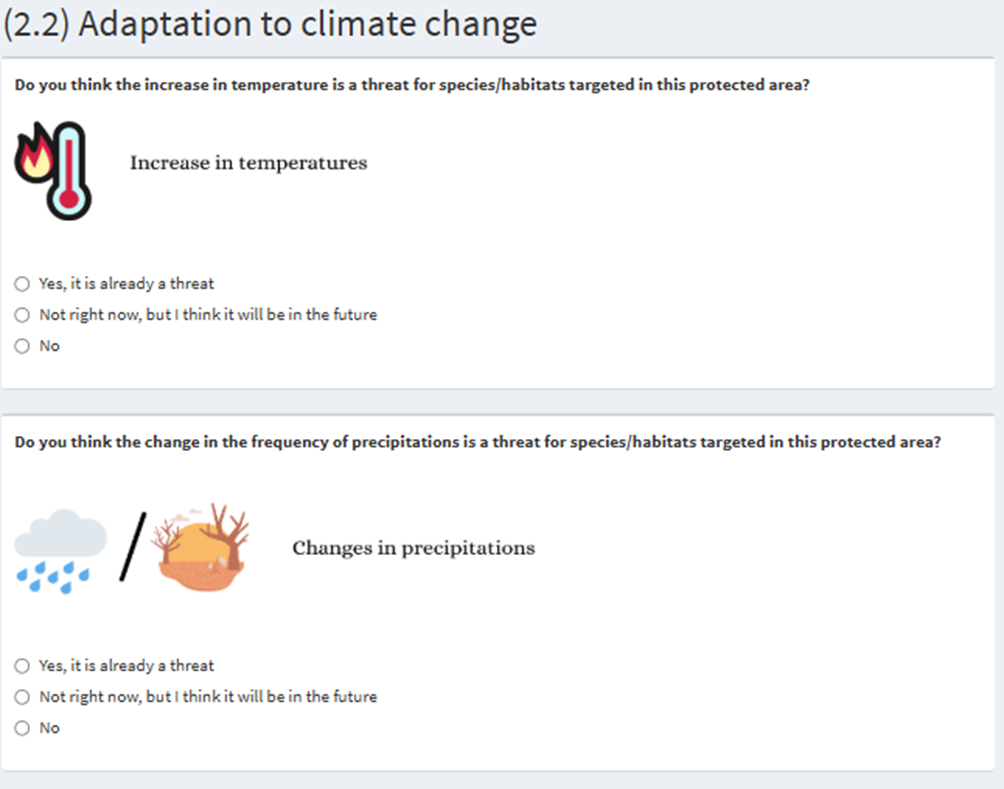

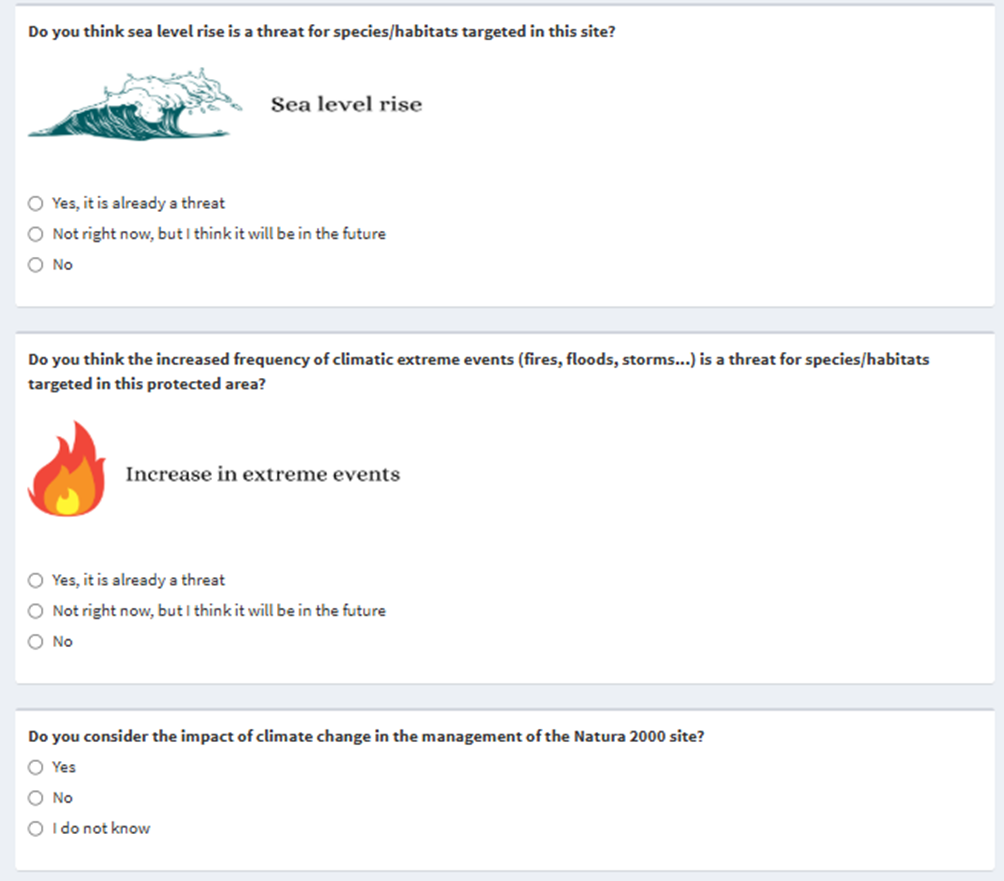

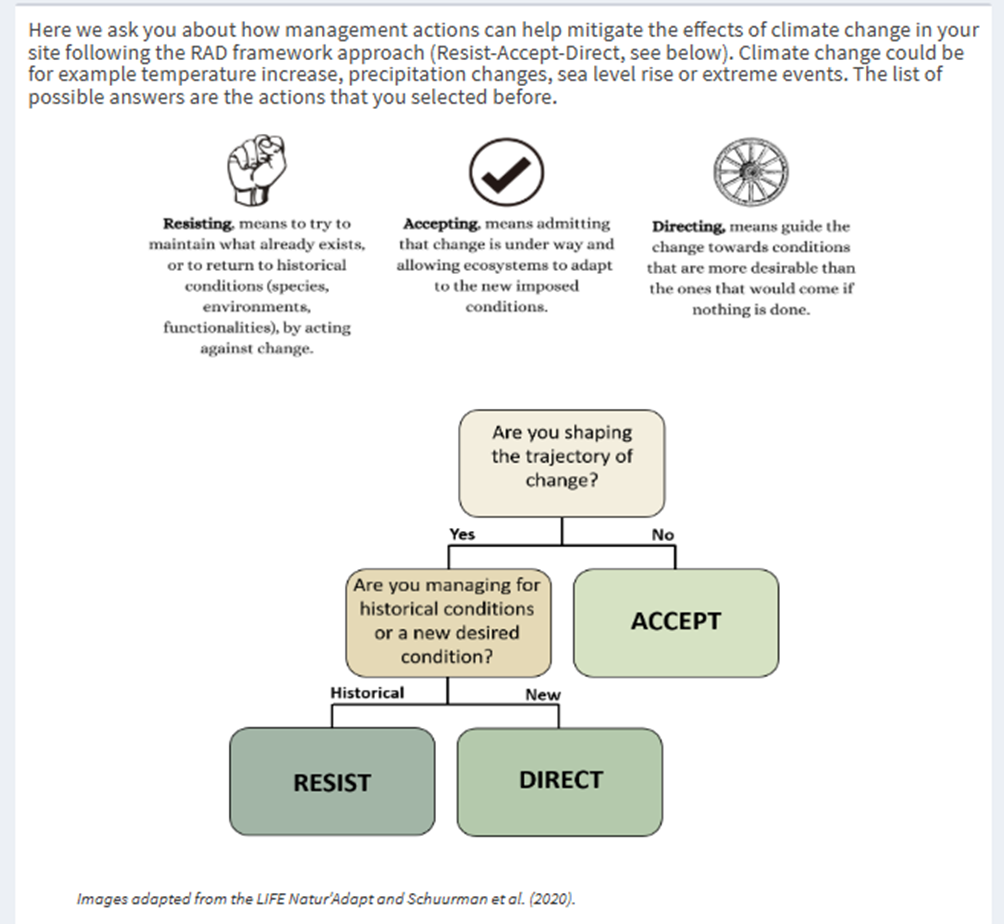

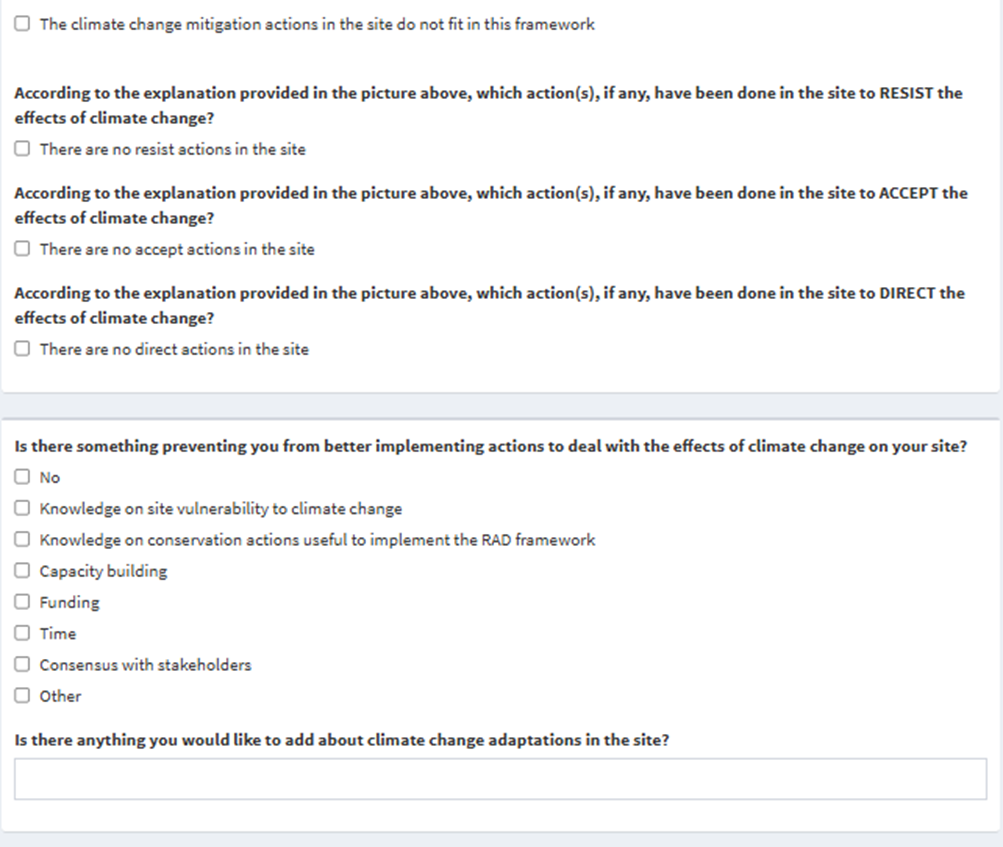

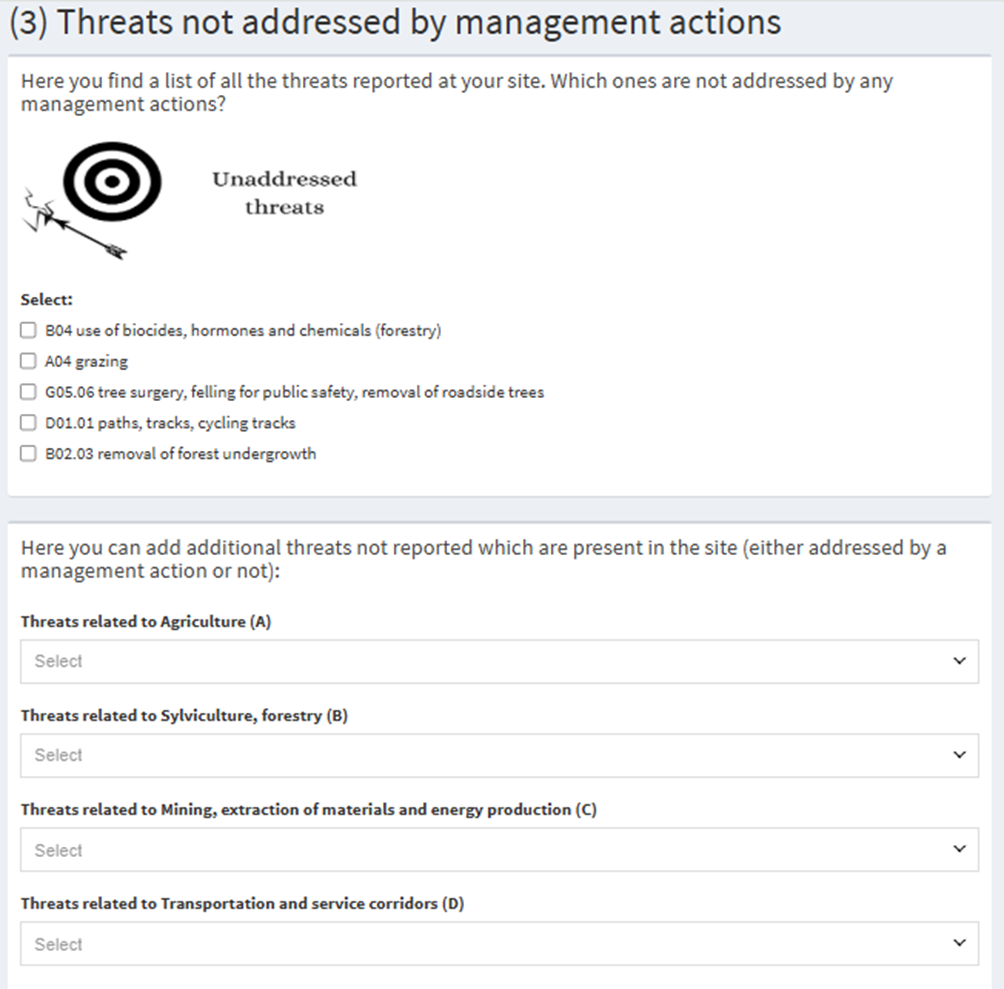

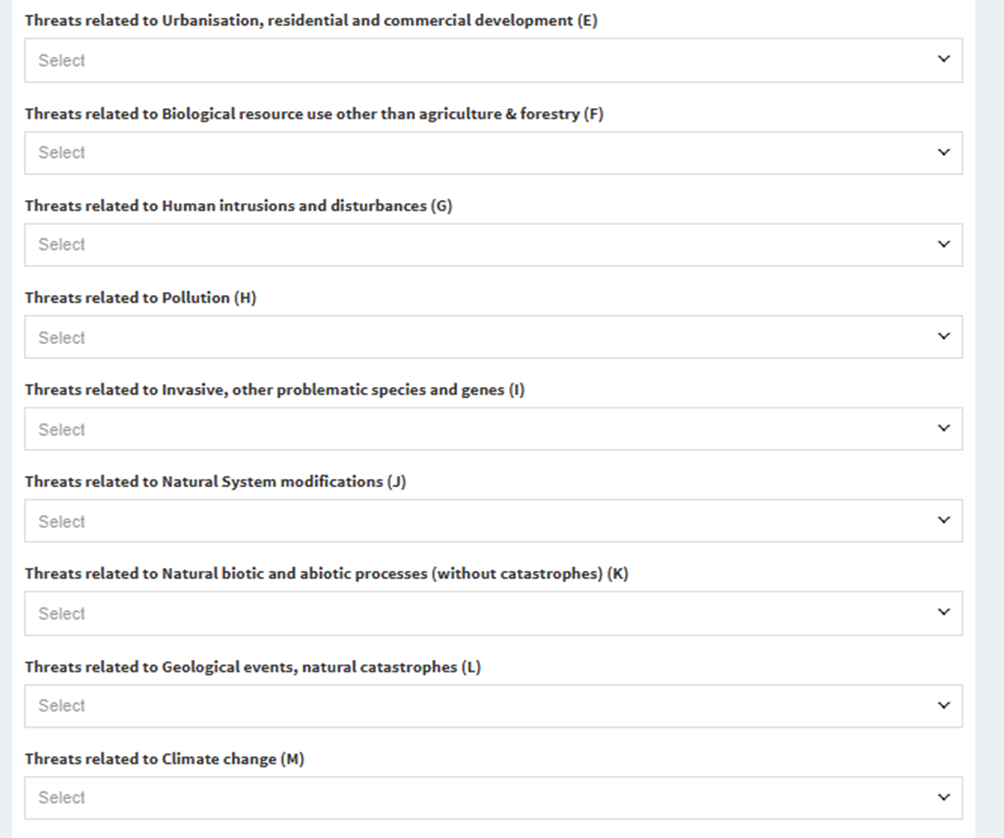

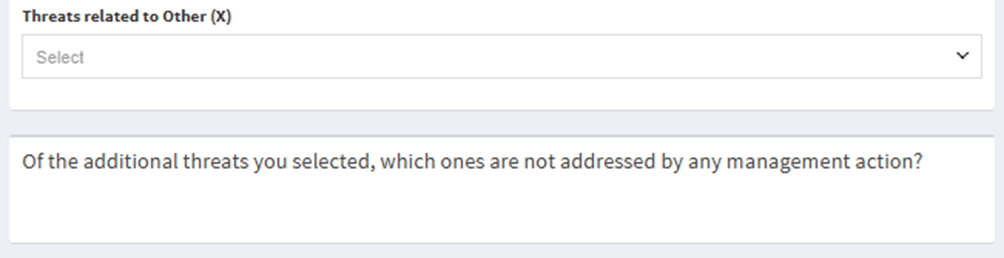

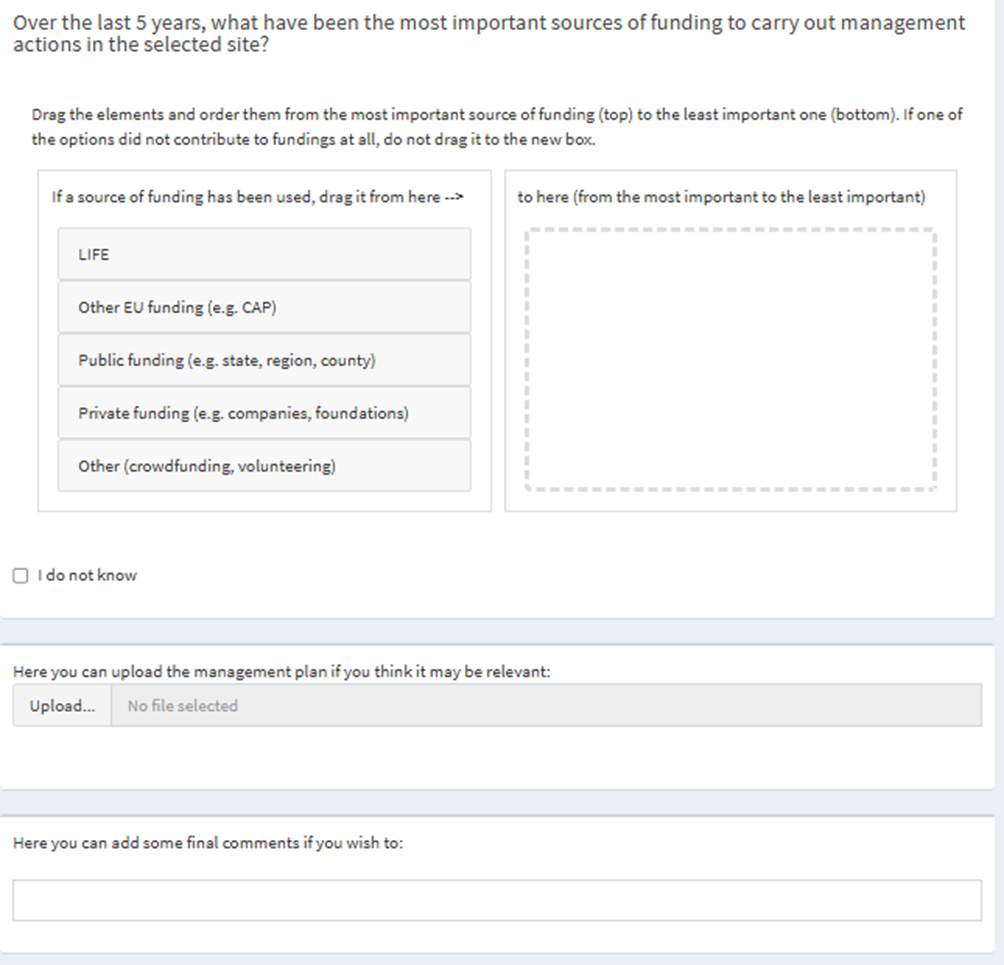
**

**Appendix S2.** Table showing the categorization we applied to the European Environmental Agency (EEA) list of conservation measures related to agriculture.

| **Conservation measure** | **Category** |
| --- | --- |
| CA01 Prevent conversion of natural and semi-natural habitats, and habitats of species into agricultural land | 1) measure preventing agricultural practices |
| CA02 Restore small landscape features on agricultural land | 1) measure preventing agricultural practices |
| CA03 Maintain existing extensive agricultural practices and agricultural landscape features | 2) measure supporting agricultural practices |
| CA04 Reinstate appropriate agricultural practices to address abandonment, including mowing, grazing, burning or equivalent measures | 2) measure supporting agricultural practices |
| CA05 Adapt mowing, grazing and other equivalent agricultural activities | 2) measure supporting agricultural practices |
| CA06 Stop mowing, grazing and other equivalent agricultural activities | 1) measure preventing agricultural practices |
| CA07 Recreate Annex I agricultural habitats | 2) measure supporting agricultural practices |
| CA08 Adapt soil management practices in agriculture | 3) measure of unspecified type related to agriculture |
| CA09 Manage the use of natural fertilisers and chemicals in agricultural (plant and animal) production | 3) measure of unspecified type related to agriculture |
| CA10 Reduce/eliminate point pollution to surface or ground waters from agricultural activities | 1) measure preventing agricultural practices |
| CA11 Reduce diffuse pollution to surface or ground waters from agricultural activities | 1) measure preventing agricultural practices |
| CA12 Reduce/eliminate air pollution from agricultural activities | 1) measure preventing agricultural practices |
| CA13 Reduce/eliminate marine pollution from agricultural activities | 1) measure preventing agricultural practices |
| CA14 Reduce/eliminate soil pollution from agricultural activities | 1) measure preventing agricultural practices |
| CA15 Manage drainage and irrigation operations and infrastructures in agriculture | 3) measure of unspecified type related to agriculture |
| CA16 Other measures related to agricultural practices | 3) measure of unspecified type related to agriculture |

**Appendix S3.** Table showing the categorization we applied to the European Environmental Agency (EEA) list of threats related to agriculture.

| **Threat** | **Category** |
| --- | --- |
| A01 Cultivation | 1) threat related to the presence of agricultural practices |
| A02 modification of cultivation practices | 1) threat related to the presence of agricultural practices |
| A02.01 agricultural intensification | 1) threat related to the presence of agricultural practices |
| A02.02 crop change | 1) threat related to the presence of agricultural practices |
| A02.03 grassland removal for arable land | 1) threat related to the presence of agricultural practices |
| A03 mowing / cutting of grassland | 3) threat of unspecified type related to agricultural practices |
| A03.01 intensive mowing or intensification | 1) threat related to the presence of agricultural practices |
| A03.02 non intensive mowing | 3) threat of unspecified type related to agricultural practices |
| A03.03 abandonment / lack of mowing | 2) threat related to the abandonment of agricultural practices |
| A04 grazing | 3) threat of unspecified type related to agricultural practices |
| A04.01 intensive grazing | 1) threat related to the presence of agricultural practices |
| A04.01.01 intensive cattle grazing | 1) threat related to the presence of agricultural practices |
| A04.01.02 intensive sheep grazing | 1) threat related to the presence of agricultural practices |
| A04.01.03 intensive horse grazing | 1) threat related to the presence of agricultural practices |
| A04.01.04 intensive goat grazing | 1) threat related to the presence of agricultural practices |
| A04.01.05 intensive mixed animal grazing | 1) threat related to the presence of agricultural practices |
| A04.02 non intensive grazing | 3) threat of unspecified type related to agricultural practices |
| A04.02.01 non intensive cattle grazing | 3) threat of unspecified type related to agricultural practices |
| A04.02.02 non intensive sheep grazing | 3) threat of unspecified type related to agricultural practices |
| A04.02.03 non intensive horse grazing | 3) threat of unspecified type related to agricultural practices |
| A04.02.04 non intensive goat grazing | 3) threat of unspecified type related to agricultural practices |
| A04.02.05 non intensive mixed animal grazing | 3) threat of unspecified type related to agricultural practices |
| A04.03 abandonment of pastoral systems, lack of grazing | 2) threat related to the abandonment of agricultural practices |
| A05 livestock farming and animal breeding (without grazing) | 3) threat of unspecified type related to agricultural practices |
| A05.01 Animal breeding | 1) threat related to the presence of agricultural practices |
| A05.02 stock feeding | 1) threat related to the presence of agricultural practices |
| A05.03 Lack of animal breeding | 2) threat related to the abandonment of agricultural practices |
| A06 annual and perennial non-timber crops | 3) threat of unspecified type related to agricultural practices |
| A06.01 annual crops for food production | 3) threat of unspecified type related to agricultural practices |
| A06.01.01 intensive annual crops for food production/ intensification | 1) threat related to the presence of agricultural practices |
| A06.01.02 non- intensive annual crops for food production | 3) threat of unspecified type related to agricultural practices |
| A06.02 perennial non-timber crops | 3) threat of unspecified type related to agricultural practices |
| A06.02.01 intensive perennial non-timber crops/intensification | 1) threat related to the presence of agricultural practices |
| A06.02.02 non-intensive perennial non-timber crops | 3) threat of unspecified type related to agricultural practices |
| A06.03 biofuel-production | 1) threat related to the presence of agricultural practices |
| A06.04 abandonment of crop production | 2) threat related to the absence of agricultural practices |
| A07 use of biocides, hormones and chemicals | 1) threat related to the presence of agricultural practices |
| A08 Fertilisation | 1) threat related to the presence of agricultural practices |
| A09 Irrigation | 1) threat related to the presence of agricultural practices |
| A10 Restructuring agricultural land holding | 1) threat related to the presence of agricultural practices |
| A10.01 removal of hedges and copses or scrub | 1) threat related to the presence of agricultural practices |
| A10.02 removal of stone walls and embankments | 1) threat related to the presence of agricultural practices |
| A11 Agriculture activities not referred to above | 3) threat of unspecified type related to agricultural practices |

**Appendix S4.** Table showing the categorization we applied to the European Nature Information System (EUNIS) habitats.

| **Habitat code** | **Category** |
| --- | --- |
| N01 | Other |
| N02 | Other |
| N03 | Other |
| N04 | Other |
| N05 | Other |
| N06 | Other |
| N07 | Other |
| N08 | Other |
| N09 | Agricultural&Grassland |
| N10 | Agricultural&Grassland |
| N11 | Agricultural&Grassland |
| N12 | Agricultural&Grassland |
| N13 | Agricultural&Grassland |
| N14 | Agricultural&Grassland |
| N15 | Agricultural&Grassland |
| N16 | Forest |
| N17 | Forest |
| N18 | Forest |
| N19 | Forest |
| N20 | Forest |
| N21 | Agricultural&Grassland |
| N22 | Other |
| N23 | Other |
| N24 | Other |
| N25 | Agricultural&Grassland |
| N26 | Forest |
| N27 | Agricultural&Grassland |

**Appendix S5**. Comparison of characteristics of the surveyed N2K sites (including spatially overlapping ones) and how they would be expected if they were a fully random subset of the whole network of existing N2K sites. a) number of observed and expected sites in each bioregion; b) number of observed and expected reported threats, divided in categories; c) distribution of observed and expected site sizes across different size categories. Significantly different comparisons are marked with * (p value < 0.05) and ** (p value < 0.001).


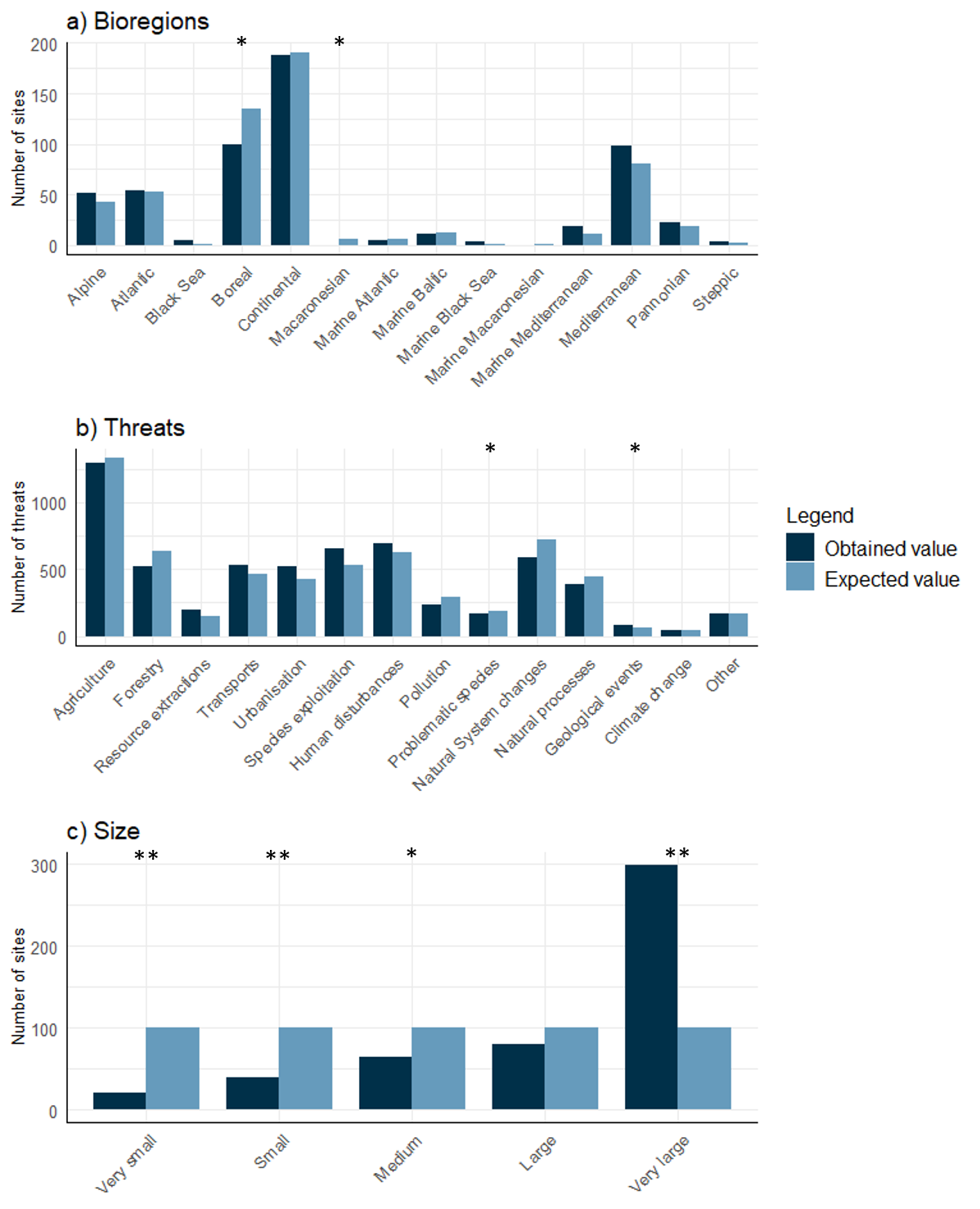


**Appendix S6.** Comprehensive pairwise comparisons results of the GLM model on conservation measures. The response variable is the presence/absence of a conservation measure in a N2K site, while the category (e.g. agriculture, forestry…etc) is the explanatory variable.

| **Contrast** | **Estimate** | **SE** | **df** | **t.ratio** | **p.value** |
| --- | --- | --- | --- | --- | --- |
| Agriculture - Climate change | 2.43 | 0.199 | 4078 | 12.231 | <0.001 |
| Agriculture - Forestry | 0.523 | 0.171 | 4078 | 3.055 | 0.094 |
| Agriculture - Human disturbance | 3.583 | 0.256 | 4078 | 14.014 | <0.001 |
| Agriculture - Natural systems and processes | 2.01 | 0.187 | 4078 | 10.734 | <0.001 |
| Agriculture - Pollution | 2.339 | 0.196 | 4078 | 11.942 | <0.001 |
| Agriculture - Problematic species | 0.824 | 0.172 | 4078 | 4.791 | <0.001 |
| Agriculture - Resource extraction | 2.652 | 0.206 | 4078 | 12.849 | <0.001 |
| Agriculture - (Species-specific measures) | 0.178 | 0.172 | 4078 | 1.032 | 0.997 |
| Agriculture - Species exploitation | 2.088 | 0.189 | 4078 | 11.042 | <0.001 |
| Agriculture - Transports | 2.936 | 0.218 | 4078 | 13.45 | <0.001 |
| Agriculture - Urbanisation | 1.737 | 0.182 | 4078 | 9.557 | <0.001 |
| Climate change - Forestry | -1.907 | 0.196 | 4078 | -9.745 | <0.001 |
| Climate change - Human disturbance | 1.153 | 0.267 | 4078 | 4.309 | 0.001 |
| Climate change - Natural systems and processes | -0.42 | 0.206 | 4078 | -2.039 | 0.666 |
| Climate change - Pollution | -0.091 | 0.213 | 4078 | -0.426 | 1 |
| Climate change - Problematic species | -1.606 | 0.195 | 4078 | -8.22 | <0.001 |
| Climate change - Resource extraction | 0.222 | 0.222 | 4078 | 0.997 | 0.998 |
| Climate change - (Species-specific measures) | -2.253 | 0.197 | 4078 | -11.418 | <0.001 |
| Climate change - Species exploitation | -0.342 | 0.208 | 4078 | -1.649 | 0.891 |
| Climate change - Transports | 0.506 | 0.233 | 4078 | 2.172 | 0.57 |
| Climate change - Urbanisation | -0.693 | 0.202 | 4078 | -3.436 | 0.029 |
| Forestry - Human disturbance | 3.059 | 0.253 | 4078 | 12.091 | <0.001 |
| Forestry - Natural systems and processes | 1.486 | 0.184 | 4078 | 8.069 | <0.001 |
| Forestry - Pollution | 1.816 | 0.193 | 4078 | 9.416 | <0.001 |
| Forestry - Problematic species | 0.301 | 0.169 | 4078 | 1.774 | 0.832 |
| Forestry - Resource extraction | 2.129 | 0.203 | 4078 | 10.464 | <0.001 |
| Forestry - (Species-specific measures) | -0.346 | 0.17 | 4078 | -2.033 | 0.671 |
| Forestry - Species exploitation | 1.564 | 0.186 | 4078 | 8.409 | <0.001 |
| Forestry - Transports | 2.413 | 0.215 | 4078 | 11.202 | <0.001 |
| Forestry - Urbanisation | 1.213 | 0.179 | 4078 | 6.788 | <0.001 |
| Human disturbance - Natural systems and processes | -1.573 | 0.26 | 4078 | -6.045 | <0.001 |
| Human disturbance - Pollution | -1.244 | 0.266 | 4078 | -4.681 | <0.001 |
| Human disturbance - Problematic species | -2.759 | 0.253 | 4078 | -10.919 | <0.001 |
| Human disturbance - Resource extraction | -0.931 | 0.273 | 4078 | -3.413 | 0.032 |
| Human disturbance - (Species-specific measures) | -3.405 | 0.254 | 4078 | -13.381 | <0.001 |
| Human disturbance - Species exploitation | -1.495 | 0.261 | 4078 | -5.721 | <0.001 |
| Human disturbance - Transports | -0.646 | 0.281 | 4078 | -2.299 | 0.477 |
| Human disturbance - Urbanisation | -1.846 | 0.257 | 4078 | -7.184 | <0.001 |
| Natural systems and processes - Pollution | 0.329 | 0.204 | 4078 | 1.618 | 0.903 |
| Natural systems and processes - Problematic species | -1.186 | 0.184 | 4078 | -6.442 | <0.001 |
| Natural systems and processes - Resource extraction | 0.642 | 0.213 | 4078 | 3.012 | 0.105 |
| Natural systems and processes - (Species-specific measures) | -1.832 | 0.186 | 4078 | -9.861 | <0.001 |
| Natural systems and processes - Species exploitation | 0.078 | 0.197 | 4078 | 0.395 | 1 |
| Natural systems and processes - Transports | 0.926 | 0.224 | 4078 | 4.129 | 0.002 |
| Natural systems and processes - Urbanisation | -0.273 | 0.191 | 4078 | -1.428 | 0.958 |
| Pollution - Problematic species | -1.515 | 0.193 | 4078 | -7.867 | <0.001 |
| Pollution - Resource extraction | 0.313 | 0.22 | 4078 | 1.421 | 0.96 |
| Pollution - (Species-specific measures) | -2.162 | 0.194 | 4078 | -11.116 | <0.001 |
| Pollution - Species exploitation | -0.251 | 0.205 | 4078 | -1.226 | 0.987 |
| Pollution - Transports | 0.597 | 0.231 | 4078 | 2.586 | 0.288 |
| Pollution - Urbanisation | -0.602 | 0.199 | 4078 | -3.025 | 0.102 |
| Problematic species - Resource extraction | 1.828 | 0.203 | 4078 | 9 | <0.001 |
| Problematic species - (Species-specific measures) | -0.646 | 0.171 | 4078 | -3.787 | 0.008 |
| Problematic species - Species exploitation | 1.264 | 0.186 | 4078 | 6.799 | <0.001 |
| Problematic species - Transports | 2.112 | 0.215 | 4078 | 9.822 | <0.001 |
| Problematic species - Urbanisation | 0.913 | 0.179 | 4078 | 5.109 | <0.001 |
| Resource extraction - (Species-specific measures) | -2.474 | 0.205 | 4078 | -12.068 | <0.001 |
| Resource extraction - Species exploitation | -0.564 | 0.215 | 4078 | -2.628 | 0.264 |
| Resource extraction - Transports | 0.284 | 0.239 | 4078 | 1.188 | 0.99 |
| Resource extraction - Urbanisation | -0.915 | 0.209 | 4078 | -4.377 | 0.001 |
| (Species-specific measures) - Species exploitation | 1.91 | 0.188 | 4078 | 10.179 | <0.001 |
| (Species-specific measures) - Transports | 2.759 | 0.217 | 4078 | 12.714 | <0.001 |
| (Species-specific measures) - Urbanisation | 1.559 | 0.18 | 4078 | 8.648 | <0.001 |
| Species exploitation - Transports | 0.848 | 0.226 | 4078 | 3.759 | 0.009 |
| Species exploitation - Urbanisation | -0.351 | 0.193 | 4078 | -1.82 | 0.807 |
| Transports - Urbanisation | -1.199 | 0.22 | 4078 | -5.44 | <0.001 |

**Appendix S7.** Comprehensive pairwise comparisons results of the GLM on unaddressed threats. The response variable is the presence/absence of an unaddressed threat in a N2K site, while its category (e.g. agriculture, forestry…etc) is the explanatory variable.

| **Contrast** | **Estimate** | **SE** | **df** | **t.ratio** | **p.value** |
| --- | --- | --- | --- | --- | --- |
| Agriculture - Climate change | 0.802 | 0.199 | 4078 | 4.023 | 0.003 |
| Agriculture - Forestry | 1.138 | 0.213 | 4078 | 5.354 | <0.001 |
| Agriculture - Human disturbance | 0.582 | 0.193 | 4078 | 3.021 | 0.103 |
| Agriculture - Natural systems and processes | -0.112 | 0.179 | 4078 | -0.626 | 1 |
| Agriculture - Other | 2.584 | 0.325 | 4078 | 7.946 | <0.001 |
| Agriculture - Pollution | 0.603 | 0.193 | 4078 | 3.12 | 0.078 |
| Agriculture - Problematic species | 1.608 | 0.238 | 4078 | 6.765 | <0.001 |
| Agriculture - Resource extraction | 1.457 | 0.229 | 4078 | 6.373 | <0.001 |
| Agriculture - Species exploitation | 0.826 | 0.2 | 4078 | 4.124 | 0.002 |
| Agriculture - Transports | 0.689 | 0.196 | 4078 | 3.519 | 0.022 |
| Agriculture - Urbanisation | 0.826 | 0.2 | 4078 | 4.124 | 0.002 |
| Climate change - Forestry | 0.336 | 0.228 | 4078 | 1.47 | 0.949 |
| Climate change - Human disturbance | -0.22 | 0.21 | 4078 | -1.047 | 0.997 |
| Climate change - Natural systems and processes | -0.914 | 0.198 | 4078 | -4.615 | <0.001 |
| Climate change - Other | 1.782 | 0.335 | 4078 | 5.311 | <0.001 |
| Climate change - Pollution | -0.199 | 0.211 | 4078 | -0.946 | 0.999 |
| Climate change - Problematic species | 0.806 | 0.252 | 4078 | 3.201 | 0.062 |
| Climate change - Resource extraction | 0.655 | 0.243 | 4078 | 2.692 | 0.23 |
| Climate change - Species exploitation | 0.024 | 0.217 | 4078 | 0.109 | 1 |
| Climate change - Transports | -0.113 | 0.213 | 4078 | -0.532 | 1 |
| Climate change - Urbanisation | 0.024 | 0.217 | 4078 | 0.109 | 1 |
| Forestry - Human disturbance | -0.556 | 0.223 | 4078 | -2.497 | 0.342 |
| Forestry - Natural systems and processes | -1.25 | 0.211 | 4078 | -5.915 | <0.001 |
| Forestry - Other | 1.446 | 0.343 | 4078 | 4.214 | 0.002 |
| Forestry - Pollution | -0.535 | 0.223 | 4078 | -2.398 | 0.407 |
| Forestry - Problematic species | 0.47 | 0.262 | 4078 | 1.794 | 0.821 |
| Forestry - Resource extraction | 0.319 | 0.254 | 4078 | 1.257 | 0.984 |
| Forestry - Species exploitation | -0.312 | 0.229 | 4078 | -1.363 | 0.97 |
| Forestry - Transports | -0.449 | 0.225 | 4078 | -1.994 | 0.698 |
| Forestry - Urbanisation | -0.312 | 0.229 | 4078 | -1.363 | 0.97 |
| Human disturbance - Natural systems and processes | -0.694 | 0.191 | 4078 | -3.629 | 0.015 |
| Human disturbance - Other | 2.002 | 0.332 | 4078 | 6.036 | <0.001 |
| Human disturbance - Pollution | 0.021 | 0.204 | 4078 | 0.102 | 1 |
| Human disturbance - Problematic species | 1.026 | 0.247 | 4078 | 4.161 | 0.002 |
| Human disturbance - Resource extraction | 0.875 | 0.238 | 4078 | 3.678 | 0.013 |
| Human disturbance - Species exploitation | 0.244 | 0.211 | 4078 | 1.155 | 0.992 |
| Human disturbance - Transports | 0.107 | 0.207 | 4078 | 0.517 | 1 |
| Human disturbance - Urbanisation | 0.244 | 0.211 | 4078 | 1.155 | 0.992 |
| Natural systems and processes - Other | 2.696 | 0.324 | 4078 | 8.309 | <0.001 |
| Natural systems and processes - Pollution | 0.715 | 0.192 | 4078 | 3.726 | 0.011 |
| Natural systems and processes - Problematic species | 1.72 | 0.237 | 4078 | 7.269 | <0.001 |
| Natural systems and processes - Resource extraction | 1.569 | 0.228 | 4078 | 6.896 | <0.001 |
| Natural systems and processes - Species exploitation | 0.938 | 0.199 | 4078 | 4.714 | <0.001 |
| Natural systems and processes - Transports | 0.801 | 0.194 | 4078 | 4.119 | 0.002 |
| Natural systems and processes - Urbanisation | 0.938 | 0.199 | 4078 | 4.714 | <0.001 |
| Other - Pollution | -1.981 | 0.332 | 4078 | -5.967 | <0.001 |
| Other - Problematic species | -0.976 | 0.359 | 4078 | -2.718 | 0.218 |
| Other - Resource extraction | -1.127 | 0.353 | 4078 | -3.19 | 0.064 |
| Other - Species exploitation | -1.758 | 0.336 | 4078 | -5.234 | <0.001 |
| Other - Transports | -1.895 | 0.333 | 4078 | -5.684 | <0.001 |
| Other - Urbanisation | -1.758 | 0.336 | 4078 | -5.234 | <0.001 |
| Pollution - Problematic species | 1.005 | 0.247 | 4078 | 4.069 | 0.003 |
| Pollution - Resource extraction | 0.854 | 0.238 | 4078 | 3.583 | 0.018 |
| Pollution - Species exploitation | 0.223 | 0.212 | 4078 | 1.054 | 0.996 |
| Pollution - Transports | 0.086 | 0.207 | 4078 | 0.414 | 1 |
| Pollution - Urbanisation | 0.223 | 0.212 | 4078 | 1.054 | 0.996 |
| Problematic species - Resource extraction | -0.151 | 0.275 | 4078 | -0.549 | 1 |
| Problematic species - Species exploitation | -0.782 | 0.252 | 4078 | -3.1 | 0.083 |
| Problematic species - Transports | -0.919 | 0.249 | 4078 | -3.692 | 0.012 |
| Problematic species - Urbanisation | -0.782 | 0.252 | 4078 | -3.1 | 0.083 |
| Resource extraction - Species exploitation | -0.631 | 0.244 | 4078 | -2.588 | 0.287 |
| Resource extraction - Transports | -0.768 | 0.24 | 4078 | -3.196 | 0.063 |
| Resource extraction - Urbanisation | -0.631 | 0.244 | 4078 | -2.588 | 0.287 |
| Species exploitation - Transports | -0.137 | 0.214 | 4078 | -0.64 | 1 |
| Species exploitation - Urbanisation | 0 | 0.218 | 4078 | 0 | 1 |
| Transports - Urbanisation | 0.137 | 0.214 | 4078 | 0.64 | 1 |

**Appendix S8.** Comprehensive pairwise comparisons results of the GLM on addressed threats. The response variable is the presence/absence of an addressed threat in a N2K site, while its category (e.g. agriculture, forestry…etc) is the explanatory variable.

| **Contrast** | **Estimate** | **SE** | **df** | **t.ratio** | **p.value** |
| --- | --- | --- | --- | --- | --- |
| Agriculture - Climate change | 2.59 | 0.194 | 4006 | 13.334 | <0.001 |
| Agriculture - Forestry | 1.263 | 0.174 | 4006 | 7.265 | <0.001 |
| Agriculture - Human disturbance | 0.712 | 0.174 | 4006 | 4.087 | 0.003 |
| Agriculture - Natural systems and processes | 0.017 | 0.182 | 4006 | 0.091 | 1 |
| Agriculture - Other | 3.234 | 0.22 | 4006 | 14.715 | <0.001 |
| Agriculture - Pollution | 1.634 | 0.176 | 4006 | 9.269 | <0.001 |
| Agriculture - Problematic species | 1.371 | 0.174 | 4006 | 7.865 | <0.001 |
| Agriculture - Resource extraction | 2.452 | 0.19 | 4006 | 12.878 | <0.001 |
| Agriculture - Species exploitation | 1.344 | 0.174 | 4006 | 7.716 | <0.001 |
| Agriculture - Transports | 1.634 | 0.176 | 4006 | 9.269 | <0.001 |
| Agriculture - Urbanisation | 1.358 | 0.174 | 4006 | 7.79 | <0.001 |
| Climate change - Forestry | -1.327 | 0.185 | 4006 | -7.189 | <0.001 |
| Climate change - Human disturbance | -1.878 | 0.186 | 4006 | -10.109 | <0.001 |
| Climate change - Natural systems and processes | -2.574 | 0.194 | 4006 | -13.269 | <0.001 |
| Climate change - Other | 0.644 | 0.227 | 4006 | 2.84 | 0.164 |
| Climate change - Pollution | -0.956 | 0.186 | 4006 | -5.133 | <0.001 |
| Climate change - Problematic species | -1.219 | 0.185 | 4006 | -6.594 | <0.001 |
| Climate change - Resource extraction | -0.138 | 0.199 | 4006 | -0.695 | 1 |
| Climate change - Species exploitation | -1.246 | 0.185 | 4006 | -6.744 | <0.001 |
| Climate change - Transports | -0.956 | 0.186 | 4006 | -5.133 | <0.001 |
| Climate change - Urbanisation | -1.232 | 0.185 | 4006 | -6.669 | <0.001 |
| Forestry - Human disturbance | -0.551 | 0.165 | 4006 | -3.342 | 0.04 |
| Forestry - Natural systems and processes | -1.247 | 0.174 | 4006 | -7.184 | <0.001 |
| Forestry - Other | 1.971 | 0.211 | 4006 | 9.338 | <0.001 |
| Forestry - Pollution | 0.37 | 0.166 | 4006 | 2.23 | 0.527 |
| Forestry - Problematic species | 0.108 | 0.164 | 4006 | 0.657 | 1 |
| Forestry - Resource extraction | 1.189 | 0.181 | 4006 | 6.583 | <0.001 |
| Forestry - Species exploitation | 0.081 | 0.164 | 4006 | 0.492 | 1 |
| Forestry - Transports | 0.37 | 0.166 | 4006 | 2.23 | 0.527 |
| Forestry - Urbanisation | 0.094 | 0.164 | 4006 | 0.575 | 1 |
| Human disturbance - Natural systems and processes | -0.696 | 0.174 | 4006 | -4 | 0.004 |
| Human disturbance - Other | 2.522 | 0.212 | 4006 | 11.884 | <0.001 |
| Human disturbance - Pollution | 0.921 | 0.167 | 4006 | 5.511 | <0.001 |
| Human disturbance - Problematic species | 0.659 | 0.165 | 4006 | 3.986 | 0.004 |
| Human disturbance - Resource extraction | 1.739 | 0.182 | 4006 | 9.571 | <0.001 |
| Human disturbance - Species exploitation | 0.632 | 0.165 | 4006 | 3.825 | 0.007 |
| Human disturbance - Transports | 0.921 | 0.167 | 4006 | 5.511 | <0.001 |
| Human disturbance - Urbanisation | 0.645 | 0.165 | 4006 | 3.905 | 0.005 |
| Natural systems and processes - Other | 3.217 | 0.22 | 4006 | 14.657 | <0.001 |
| Natural systems and processes - Pollution | 1.617 | 0.176 | 4006 | 9.192 | <0.001 |
| Natural systems and processes - Problematic species | 1.355 | 0.174 | 4006 | 7.784 | <0.001 |
| Natural systems and processes - Resource extraction | 2.435 | 0.19 | 4006 | 12.812 | <0.001 |
| Natural systems and processes - Species exploitation | 1.328 | 0.174 | 4006 | 7.635 | <0.001 |
| Natural systems and processes - Transports | 1.617 | 0.176 | 4006 | 9.192 | <0.001 |
| Natural systems and processes - Urbanisation | 1.341 | 0.174 | 4006 | 7.71 | <0.001 |
| Other - Pollution | -1.6 | 0.213 | 4006 | -7.529 | <0.001 |
| Other - Problematic species | -1.863 | 0.211 | 4006 | -8.816 | <0.001 |
| Other - Resource extraction | -0.782 | 0.224 | 4006 | -3.499 | 0.024 |
| Other - Species exploitation | -1.89 | 0.211 | 4006 | -8.948 | <0.001 |
| Other - Transports | -1.6 | 0.213 | 4006 | -7.529 | <0.001 |
| Other - Urbanisation | -1.876 | 0.211 | 4006 | -8.882 | <0.001 |
| Pollution - Problematic species | -0.263 | 0.166 | 4006 | -1.577 | 0.917 |
| Pollution - Resource extraction | 0.818 | 0.182 | 4006 | 4.486 | <0.001 |
| Pollution - Species exploitation | -0.29 | 0.166 | 4006 | -1.741 | 0.849 |
| Pollution - Transports | 0 | 0.168 | 4006 | 0 | 1 |
| Pollution - Urbanisation | -0.276 | 0.166 | 4006 | -1.659 | 0.886 |
| Problematic species - Resource extraction | 1.081 | 0.181 | 4006 | 5.975 | <0.001 |
| Problematic species - Species exploitation | -0.027 | 0.165 | 4006 | -0.165 | 1 |
| Problematic species - Transports | 0.263 | 0.166 | 4006 | 1.577 | 0.917 |
| Problematic species - Urbanisation | -0.014 | 0.165 | 4006 | -0.082 | 1 |
| Resource extraction - Species exploitation | -1.108 | 0.181 | 4006 | -6.128 | <0.001 |
| Resource extraction - Transports | -0.818 | 0.182 | 4006 | -4.486 | <0.001 |
| Resource extraction - Urbanisation | -1.094 | 0.181 | 4006 | -6.052 | <0.001 |
| Species exploitation - Transports | 0.29 | 0.166 | 4006 | 1.741 | 0.849 |
| Species exploitation - Urbanisation | 0.014 | 0.164 | 4006 | 0.082 | 1 |
| Transports - Urbanisation | -0.276 | 0.166 | 4006 | -1.659 | 0.886 |

**Appendix S9.** Percentage distribution of threat categories among selected threats not currently reported in the Standard Data Forms (SDF) of the surveyed Natura 2000 sites.

| **Threat category** | **Percentage (%)** |
| --- | --- |
| Agriculture (A) | 14.02 |
| Biological resource use other than agriculture & forestry (F) | 4.24 |
| Climate change (M) | 15.67 |
| Geological events, natural catastrophes (L) | 2.79 |
| Human intrusions and disturbances (G) | 8.44 |
| Invasive, other problematic species and genes (I) | 4.40 |
| Mining, extraction of materials and energy production (C) | 2.20 |
| Natural biotic and abiotic processes (without catastrophes) (K) | 11.67 |
| Natural System modifications (J) | 12.80 |
| Other (X) | 0.71 |
| Pollution (H) | 9.90 |
| Sylviculture, forestry (B) | 5.11 |
| Transportation and service corridors (D) | 3.65 |
| Urbanisation, residential and commercial development € | 4.40 |

**Appendix S10.** Tables showing percentages of agricultural actions selected as main actions in N2K sites dominated by three different habitat types:

1. Grassland and agricultural sites

| **Measure** | **Type** | **Percentage (%)** |
| --- | --- | --- |
| CA01 Prevent conversion of natural and semi-natural habitats, and habitats of species into agricultural land | Measures preventing agricultural practices | 1.1 |
| CA02 Restore small landscape features on agricultural land | Measures preventing agricultural practices | 0 |
| CA03 Maintain existing extensive agricultural practices and agricultural landscape features | Measures supporting agricultural practices | 22.8 |
| CA04 Reinstate appropriate agricultural practices to address abandonment, including mowing, grazing, burning or equivalent measures | Measures supporting agricultural practices | 19.6 |
| CA05 Adapt mowing, grazing and other equivalent agricultural activities | Measures supporting agricultural practices | 10.9 |
| CA06 Stop mowing, grazing and other equivalent agricultural activities | Measures preventing agricultural practices | 1.1 |
| CA07 Recreate Annex I agricultural habitats | Measures supporting agricultural practices | 5.4 |
| CA08 Adapt soil management practices in agriculture | Measures of unspecified type related to agriculture | 0 |
| CA09 Manage the use of natural fertilisers and chemicals in agricultural (plant and animal) production | Measures of unspecified type related to agriculture | 0 |
| CA10 Reduce/eliminate point pollution to surface or ground waters from agricultural activities | Measures preventing agricultural practices | 0 |
| CA11 Reduce diffuse pollution to surface or ground waters from agricultural activities | Measures preventing agricultural practices | 0 |
| CA12 Reduce/eliminate air pollution from agricultural activities | Measures preventing agricultural practices | 0 |
| CA13 Reduce/eliminate marine pollution from agricultural activities | Measures preventing agricultural practices | 0 |
| CA14 Reduce/eliminate soil pollution from agricultural activities | Measures preventing agricultural practices | 0 |
| CA15 Manage drainage and irrigation operations and infrastructures in agriculture | Measures of unspecified type related to agriculture | 1.1 |
| CA16 Other measures related to agricultural practices | Measures of unspecified type related to agriculture | 0 |

1. Forest sites

| **Measure** | **Type** | **Percentage (%)** |
| --- | --- | --- |
| CA01 Prevent conversion of natural and semi-natural habitats, and habitats of species into agricultural land | Measures preventing agricultural practices | 1.6 |
| CA02 Restore small landscape features on agricultural land | Measures preventing agricultural practices | 0.8 |
| CA03 Maintain existing extensive agricultural practices and agricultural landscape features | Measures supporting agricultural practices | 8.7 |
| CA04 Reinstate appropriate agricultural practices to address abandonment, including mowing, grazing, burning or equivalent measures | Measures supporting agricultural practices | 11 |
| CA05 Adapt mowing, grazing and other equivalent agricultural activities | Measures supporting agricultural practices | 7.9 |
| CA06 Stop mowing, grazing and other equivalent agricultural activities | Measures preventing agricultural practices | 0 |
| CA07 Recreate Annex I agricultural habitats | Measures supporting agricultural practices | 6.3 |
| CA08 Adapt soil management practices in agriculture | Measures of unspecified type related to agriculture | 0 |
| CA09 Manage the use of natural fertilisers and chemicals in agricultural (plant and animal) production | Measures of unspecified type related to agriculture | 0 |
| CA10 Reduce/eliminate point pollution to surface or ground waters from agricultural activities | Measures preventing agricultural practices | 0 |
| CA11 Reduce diffuse pollution to surface or ground waters from agricultural activities | Measures preventing agricultural practices | 0 |
| CA12 Reduce/eliminate air pollution from agricultural activities | Measures preventing agricultural practices | 0 |
| CA13 Reduce/eliminate marine pollution from agricultural activities | Measures preventing agricultural practices | 0 |
| CA14 Reduce/eliminate soil pollution from agricultural activities | Measures preventing agricultural practices | 0 |
| CA15 Manage drainage and irrigation operations and infrastructures in agriculture | Measures of unspecified type related to agriculture | 0 |
| CA16 Other measures related to agricultural practices | Measures of unspecified type related to agriculture | 0.8 |

1. Other sites

| **Measure** | **Type** | **Percentage (%)** |
| --- | --- | --- |
| CA01 Prevent conversion of natural and semi-natural habitats, and habitats of species into agricultural land | Measures preventing agricultural practices | 1.7 |
| CA02 Restore small landscape features on agricultural land | Measures preventing agricultural practices | 0.8 |
| CA03 Maintain existing extensive agricultural practices and agricultural landscape features | Measures supporting agricultural practices | 10.8 |
| CA04 Reinstate appropriate agricultural practices to address abandonment, including mowing, grazing, burning or equivalent measures | Measures supporting agricultural practices | 10 |
| CA05 Adapt mowing, grazing and other equivalent agricultural activities | Measures supporting agricultural practices | 6.7 |
| CA06 Stop mowing, grazing and other equivalent agricultural activities | Measures preventing agricultural practices | 0 |
| CA07 Recreate Annex I agricultural habitats | Measures supporting agricultural practices | 5 |
| CA08 Adapt soil management practices in agriculture | Measures of unspecified type related to agriculture | 0 |
| CA09 Manage the use of natural fertilisers and chemicals in agricultural (plant and animal) production | Measures of unspecified type related to agriculture | 0 |
| CA10 Reduce/eliminate point pollution to surface or ground waters from agricultural activities | Measures preventing agricultural practices | 0 |
| CA11 Reduce diffuse pollution to surface or ground waters from agricultural activities | Measures preventing agricultural practices | 0 |
| CA12 Reduce/eliminate air pollution from agricultural activities | Measures preventing agricultural practices | 0 |
| CA13 Reduce/eliminate marine pollution from agricultural activities | Measures preventing agricultural practices | 0 |
| CA14 Reduce/eliminate soil pollution from agricultural activities | Measures preventing agricultural practices | 0 |
| CA15 Manage drainage and irrigation operations and infrastructures in agriculture | Measures of unspecified type related to agriculture | 0 |
| CA16 Other measures related to agricultural practices | Measures of unspecified type related to agriculture | 1.7 |

**Appendix S11.** Comprehensive results of the three GLMs testing for differences in the main funding source used in sites where the most important action for biodiversity is related to agriculture and not. A different model was run for each of the predominant funding sources (EU including CAP, public funding, LIFE), using data on presence/absence of the funding source as response variable and binary data on sites with or without the main action related to agriculture as explanatory variable.

| **Model** | **estimate** | **SE** | **df** | **t.ratio** | **p.value** |
| --- | --- | --- | --- | --- | --- |
| Public funding | -0.498 | 0.314 | 324 | -1.585 | 0.1141 |
| CAP and other EU funding | 1.005 | 0.339 | 324 | 2.967 | 0.0032 |
| LIFE funding | -0.760 | 0.471 | 324 | -1.612 | 0.1079 |
